# Controlling Coulomb correlations and fine structure of quasi-one-dimensional excitons by magnetic order

**DOI:** 10.1038/s41563-025-02120-1

**Published:** 2025-02-19

**Authors:** M. Liebich, M. Florian, N. Nilforoushan, F. Mooshammer, A. D. Koulouklidis, L. Wittmann, K. Mosina, Z. Sofer, F. Dirnberger, M. Kira, R. Huber

**Affiliations:** 1 Department of Physics, University of Regensburg, Regensburg, Germany; 2 Department of Electrical Engineering and Computer Science, University of Michigan, Ann Arbor, MI USA; 3 Regensburg Center for Ultrafast Nanoscopy, University of Regensburg, Regensburg, Germany; 4 Department of Inorganic Chemistry, University of Chemistry and Technology Prague, Prague, Czech Republic; 5 Institute of Applied Physics, Dresden University of Technology, Dresden, Germany; 6 Present Address: Université Paris Cité, CNRS, Laboratoire Matériaux et Phénomènes Quantiques, Paris, France; 7 Present Address: Department of Physics, Technical University of Munich, Munich, Germany

**Keywords:** Ultrafast photonics, Magnetic properties and materials, Two-dimensional materials, Two-dimensional materials

## Abstract

Many surprising properties of quantum materials result from Coulomb correlations defining electronic quasiparticles and their interaction chains. In van der Waals layered crystals, enhanced correlations have been tailored in reduced dimensions, enabling excitons with giant binding energies and emergent phases including ferroelectric, ferromagnetic and multiferroic orders. Yet, correlation design has primarily relied on structural engineering. Here we present quantitative experiment–theory proof that excitonic correlations can be switched through magnetic order. By probing internal Rydberg-like transitions of excitons in the magnetic semiconductor CrSBr, we reveal their binding energy and a dramatic anisotropy of their quasi-one-dimensional orbitals manifesting in strong fine-structure splitting. We switch the internal structure from strongly bound, monolayer-localized states to weakly bound, interlayer-delocalized states by pushing the system from antiferromagnetic to paramagnetic phases. Our analysis connects this transition to the exciton’s spin-controlled effective quantum confinement, supported by the exciton’s dynamics. In future applications, excitons or even condensates may be interfaced with spintronics; extrinsically switchable Coulomb correlations could shape phase transitions on demand.

## Abstract (web summary)

The antiferromagnetic-to-paramagnetic phase transition in a two-dimensional semiconducting magnet, CrSBr, induces an exciton confinement transition from a strongly bound quasi-one-dimensional state to a weakly bound interlayer-delocalized state.

## Main

Atomically thin van der Waals (vdW) crystals^1^ have formed a paragon of how Coulomb correlations can be dramatically enhanced by quantum confinement and reduced dielectric screening. Excitons—Coulomb-bound electron–hole pairs—have reached binding energies of hundreds of millielectronvolts^2^. Correlations have been precisely controlled by atomic-level structural engineering, including layer stacking^3–6^ and twist-angle control^7–9^, which has given rise to superconductivity^10^, topological phases^11^ and many other fascinating phenomena^12^ in vdW materials. In one-dimensional (1D) systems, the interaction among electrons is qualitatively different from their higher-dimensional counterparts, as prototypically described by the Tomonaga–Luttinger liquid theory^13^. Besides even stronger Coulomb interaction^14,15^, 1D systems can feature exotic phenomena, such as 1D spin chains^16^, anomalous quantum tunnelling^17^ and spin-charge separation^18^. Yet, comprehensive in situ tuning of the properties of 1D systems has remained challenging.

In this context, the advent of magnetic vdW materials^19,20^, featuring intertwined excitonic correlations and spin degrees of freedom, has provided new prospects for the field of quantum materials^21^. These crystals promise flexible control over Coulomb correlations via the magnetic order that is orders of magnitude more efficient even than, for instance, prominent Zeeman shifts in transition metal dichalcogenides^22–24^. In particular, the recently rediscovered vdW magnet CrSBr distinguishes itself through its magneto-optical properties resulting from quasi-1D excitons coupled to A-type antiferromagnetism^25,26^. Interband spectroscopy has found convincing evidence for the magnetic order to influence interlayer hybridization^25–27^ and, thus, the interband resonances of optically bright excitons without the need to change the structure itself. Yet, interband spectroscopy cannot directly distinguish Coulomb-correlation effects from single-particle bandgap renormalization^28^, which can also dramatically change across a phase transition^25^. Moreover, the feeble oscillator strength for light emission polarized along the crystal *a* axis^26^ complicates conclusions about the internal structure of excitons in CrSBr.

Here we use mid-infrared (MIR) excitonic Rydberg spectroscopy^6,9,29–32^, which is independent of interband selection rules and can directly and exclusively probe internal exciton transitions^33^, shaped entirely by Coulomb correlations. Combining these measurements with state-of-the-art many-body theory^26,28,34,35^, we can quantitatively resolve Coulomb correlation effects across the transition from an antiferromagnetic (AFM) to a paramagnetic (PM) state in CrSBr. The anisotropy-induced fine structure of all optically dark and bright excitons reveals their binding energy and the quasi-1D nature of the exciton orbitals. Most importantly, the AFM–PM phase transition strongly modifies the internal exciton structure and, thus, the underlying Coulomb correlations. Our theory explains this behaviour by strongly different spin restrictions of the out-of-plane quantum confinement in both phases, enabling efficient extrinsic control of dimensionality and Coulomb correlations. This scenario is confirmed by the temperature dependence of the ultrafast exciton formation and decay dynamics.

## Revealing the internal fine structure of quasi-1D excitons

**Fig. 1:**
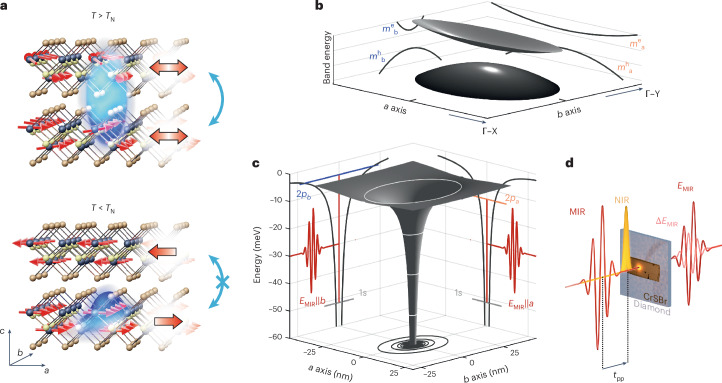
Accessing the internal structure of anisotropic excitons in CrSBr. **a**, Schematic of the coupling of excitons (blue surfaces) to the magnetic order (red arrows) in CrSBr (dark spheres, Cr; yellow spheres, S; brown spheres, Br). In the AFM phase (*T* < *T*_N_, large red arrows), the quasi-1D excitons stretched along the *b* axis are confined to individual layers (bottom) because interlayer hopping is spin-forbidden (crossed-out blue double arrow). The paramagnetic phase (*T* > *T*_N_, large red double arrows) allows excitons to be delocalized (blue double arrow) across neighbouring layers (top). **b**, Schematic of the band structure of CrSBr. For clarity, the bandgap is substantially reduced. The projections of the elliptic paraboloids onto the *a* (Γ–X) and *b* (Γ–Y) directions highlight the large difference in the effective mass of the lowest conduction band (*m*^e^) and the highest valence band (*m*^h^). **c**, Anisotropic Coulomb potential (grey surface) of the excitons. The isoenergy contours (white ellipses) and the projections (black lines) onto the two crystallographic directions highlight the large in-plane anisotropy. Although the *a* and *b* axes share a common 1*s* level (light grey line), the degeneracy of the 2*p* states is lifted. Consequently, the 1*s*–2*p*_*a*_ and 1*s*–2*p*_*b*_ transitions (vertical red lines) feature different energies that are interrogated by MIR transients (red waveforms) of orthogonal polarization (*E*_MIR_||*a* and *E*_MIR_||*b*, respectively). **d**, Schematic of the time-resolved NIR pump–MIR probe spectroscopy experiment. After a variable delay time *t*_pp_, the electric field of the MIR waveform is transmitted through the CrSBr crystal and the diamond substrate. The transmitted MIR transient, *E*_MIR_, and changes to its electric field, Δ*E*_MIR_, induced by the presence of the photogenerated electron–hole pairs are electro-optically recorded.

In CrSBr, chromium and sulfur atoms form 1D chains along the crystallographic *b* axis^36^ (Fig. 1a). This gives rise to an anisotropic band structure with dramatically different effective masses along the *a* (Γ–X) and *b* (Γ–Y) directions (Fig. 1b). For the conduction band minimum, the corresponding values are ${m}_{{a}}^{{\rm{e}}}=7.31\times {m}_{0}$ and ${m}_{{b}}^{{\rm{e}}}=0.14\times {m}_{0}$ (ref. ^26^), where *m*_0_ is the bare electron mass, leading to a ratio of ${m}_{{a}}^{{\rm{e}}}/{m}_{{b}}^{{\rm{e}}}=52$. The structural anisotropy also leads to markedly different dielectric functions along the *a* and *b* axes, with *ε*_*b*_ ≫ *ε*_*a*_ (refs. ^26,27^), which entails a strongly asymmetric Coulomb potential (Fig. 1c). This combined mass and Coulombic asymmetry is expected to create strongly anisotropic excitons whose wavefunctions mainly extend along the *b* axis with a lifted degeneracy of energy levels, as explained in the next section. The effective quantum confinement of these excitons sensitively depends on whether electrons can spread over neighbouring monolayers. Below the Néel temperature^37^ (*T*_N_ = 132 K), CrSBr exhibits in-plane ferromagnetically ordered layers, which—in the bulk—are antiferromagnetically coupled to adjacent layers. This coupling mechanism blocks the interlayer hopping^25^ of electron–hole pairs and, consequently, confines the Coulomb-bound pairs in one dimension within individual layers^26^ (Fig. 1a, bottom). Nevertheless, elevating the temperature through a potential intermediate coexistence regime of ferromagnetic (FM) and AFM orders^38,39^ to the PM phase should enable interlayer hybridization, relax the strict out-of-plane confinement and impart a less 1D character on the excitons (Fig. 1a, top).

**Extended Data Fig. 1:**
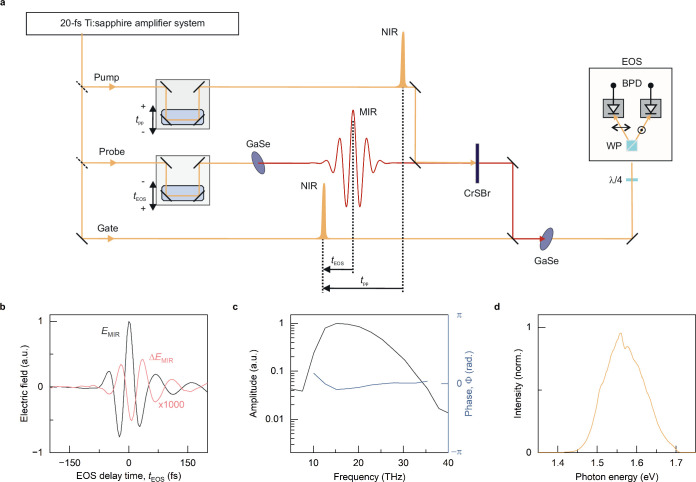
NIR pump–MIR probe setup. **a**, Schematic of the setup. Ultrashort NIR pulses delivered from a Ti:sapphire amplifier system are split into three branches. ‘Pump’: a variable delay time *t*_pp_ between NIR pump and MIR probe pulses allows for studying the non-equilibrium dynamics on a femtosecond timescale. ‘Probe’: MIR probe pulses are generated by optical rectification in a gallium selenide crystal (GaSe). ‘Gate’: electro-optic sampling is employed to record the MIR time-domain waveform in a second nonlinear GaSe crystal as a function of the electro-optic delay time *t*_EOS_. **b**, Electro-optically detected MIR waveform *E*_MIR_ (grey) of the probe pulse and pump-induced change of the probe electric field Δ*E*_MIR_ (light pink) as a function of the electro-optic sampling time *t*_EOS_. **c**, Corresponding amplitude spectrum (grey) and spectral phase (blue) of the MIR probe pulse as a function of the frequency retrieved by Fourier transformation. **d**, Spectrum of the NIR pump pulses from the Ti:sapphire amplifier system.

To explore the internal structure and the dynamics of excitons in CrSBr, we first prepare free electron–hole pairs in the AFM phase of a 620-nm-thick bulk sample using optical excitation across the single-particle bandgap with femtosecond near-infrared (NIR) pulses (centre wavelength, 785 nm; pulse duration, 20 fs; Fig. 1d and Extended Data Fig. 1). The polarization is set along the *b* axis to ensure effective excitation utilizing the large transition dipole moment in this direction as opposed to the *a* axis^25,26^. Following above-bandgap excitation, excitons form on a subpicosecond timescale, as discussed later. After a variable delay time *t*_pp_, a phase-locked MIR pulse is transmitted through the sample to interrogate intraexcitonic transitions (Fig. 1c,d, red waveforms), for example, between the 1*s* and 2*p* orbitals^6,9,29,32^. By simultaneously recording the electric field of the MIR waveform, *E*_MIR_, and its pump-induced change, Δ*E*_MIR_, using electro-optic sampling, we can access the full dielectric function of the photoexcited electron–hole ensemble in the MIR regime^40^ and retrieve the internal structure of excitons^32^ (Methods). By rotating the MIR electric field polarization, we can selectively probe the response of the photoexcited electron–hole pairs along the *a* or *b* axis (Fig. 1c). The elongated shape^36,38^ of the exfoliated CrSBr flake indicates its crystallographic orientation (Fig. 1d and Supplementary Fig. 1).

**Fig. 2:**
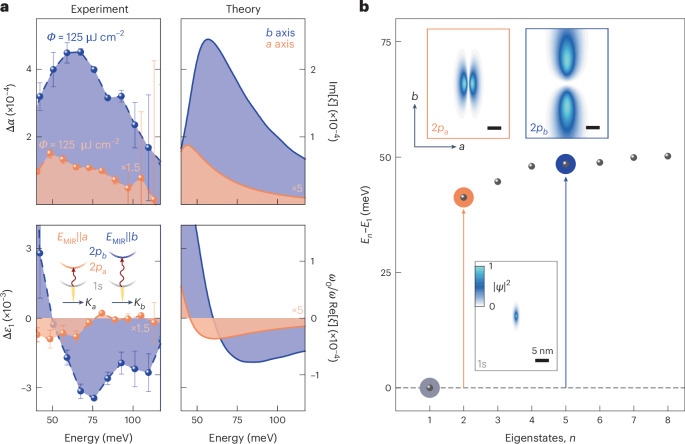
Fine structure of quasi-1D excitons in CrSBr. **a**, Experimental (left) and theoretical (right) pump-induced changes in the MIR absorption (Δ*α*; top) and the real part of the dielectric function (Δ*ε*_1_; bottom) as a function of photon energy. The experimental data were recorded at a pump–probe delay time of *t*_pp_ = 500 fs along the crystallographic *a* (orange dots; pump fluence, *Φ*_NIR_ = 450 μJ cm^–2^) and *b* (blue dots; *Φ*_NIR_ = 125 μJ cm^–2^) axes and at a lattice temperature of *T* = 5 K. The error bars were retrieved by quantifying the uncertainty in the pump-induced change to the electric field Δ*E*_MIR_ via the standard deviation of a set of five measurements. For clarity, the experimental and theoretical data along the *a* axis have been scaled by a factor of 1.5 and 5, respectively. The rather large fluence needed to achieve a measurable MIR response along the *a* axis indicates a weaker intraexcitonic oscillator strength compared with that along the *b* axis. Our experiment–theory analysis is fully quantitative since Rydberg spectroscopy measures changes in the transmitted MIR field that have a one-to-one connection to a uniquely scaled susceptibility^32,33^ *ξ* (right axis). This allows us to easily construct the intrinsic linear absorption *α* = 2Im[*ξ*] and Δ*ε*_1_ (Methods). Inset: schematic of the internal quantum transitions of anisotropic photogenerated excitons with non-degenerate 2*p* states triggered by MIR pulses polarized along the crystallographic *a* or *b* axis (*E*_MIR_||*a* or *E*_MIR_||*b*, respectively). The dispersions are plotted as a function of centre-of-mass momentum *K*. **b**, Calculated series of eigenenergies *E*_*n*_ relative to the ground-state energy *E*_1_. The degeneracy of the 2*p* states is lifted resulting in a splitting of ~13 meV. The arrows indicate the transition energy from the 1*s* state (horizontal dashed lines) to the energetically higher-lying 2*p*_*a*_ (orange) and 2*p*_*b*_ (blue) states. Insets: probability densities |*Ψ*|^2^ for 1*s* (grey box), 2*p*_*a*_ (orange box) and 2*p*_*b*_ (blue box) orbitals of a quasi-1D exciton derived from the anisotropic effective mass and dielectric screening (compare Fig. 1b,c; Methods).

Figure 2a shows the pump-induced changes in the MIR absorption Δ*α* and the real part of the dielectric function Δ*ε*_1_ retrieved from the time-domain data at a pump–probe delay time of *t*_pp_ = 500 fs after photoexcitation and a lattice temperature of *T* = 5 K. When *E*_MIR_ is polarized along the *b* direction (Fig. 2a, blue spheres), Δ*α* features a pronounced maximum at a photon energy of 62 meV, whereas Δ*ε*_1_ exhibits a dispersive shape at nearly the same energy. Minor spectral shifts between the two features are expected from residual electron–hole plasma contributions.

We analyse our measurements with a many-body theory that comprehensively incorporates band structure details, asymmetric dielectric screening of the Coulomb interaction and polaritonic effects (Methods). Our computations (Fig. 2a, right) simultaneously confirm the resonant peak in Δ*α* and the dispersive shape in Δ*ε*_1_, and assign them to a Rydberg-like intraexcitonic transition between the 1*s* exciton and 2*p*_*b*_ state. Higher-lying *p* states yield an asymmetric line shape, whereas the 1*s* state is slightly shifted (by 10 meV) owing to its polaritonic nature^27^ at NIR frequencies (Methods). In stark contrast, probing with *E*_MIR_||*a* yields a substantially reduced response with a resonance that is redshifted by 13 meV, in both experiment (Fig. 2a, orange spheres) and theory (Fig. 2a, right). Thus, the 1*s*–2*p*_*a*_ transition is located at lower energies than the 1*s*–2*p*_*b*_ transition, proving that the degeneracy of the 2*p* states, observed in isotropic systems^6,29^, is strongly lifted in CrSBr. To the best of our knowledge, this is the first observation of excitonic fine structure made by direct Rydberg spectroscopy.

To quantitatively test our assignment of resonances, we compute the exciton wavefunctions and their corresponding fine structure. The lowest-energy exciton states are shown in Fig. 2b (insets) together with the Rydberg series (dots). The 1*s* state is spatially confined on the single-nanometre scale and is extremely anisotropic, featuring a Bohr radius of 0.7 nm and 2.5 nm along the *a* and *b* axes, respectively. The strong anisotropy also gives rise to drastically different 2*p*_*a*_ and 2*p*_*b*_ states, which exhibit a characteristic sign change along the *a* and *b* axes, respectively. Whereas the 2*p*_*a*_ state is strongly curved with a Bohr radius of 2.8 nm along the *a* axis, the 2*p*_*b*_ orbital is much more elongated (17.2 nm) along the *b* axis. The large average distance between the electron and hole reduces the binding energy of the 2*p*_*b*_ orbital compared with the 2*p*_*a*_ state by 13 meV, as seen in the series of eigenenergies depicted in Fig. 2b. This picture also explains the strong anisotropy in the magnitude of the MIR response (Fig. 2a). The 1*s* and 2*p*_*b*_ states are both elongated along the *b* axis, resulting in a large oscillator strength. Conversely, the 1*s* and 2*p*_*a*_ states are aligned along nearly orthogonal directions, which suppresses the transition rate by approximately a factor of five, consistently proving the exceptional in-plane anisotropy and almost-1D nature of excitons in CrSBr.

## Magnetic-order control of excitonic quantum confinement

**Fig. 3:**
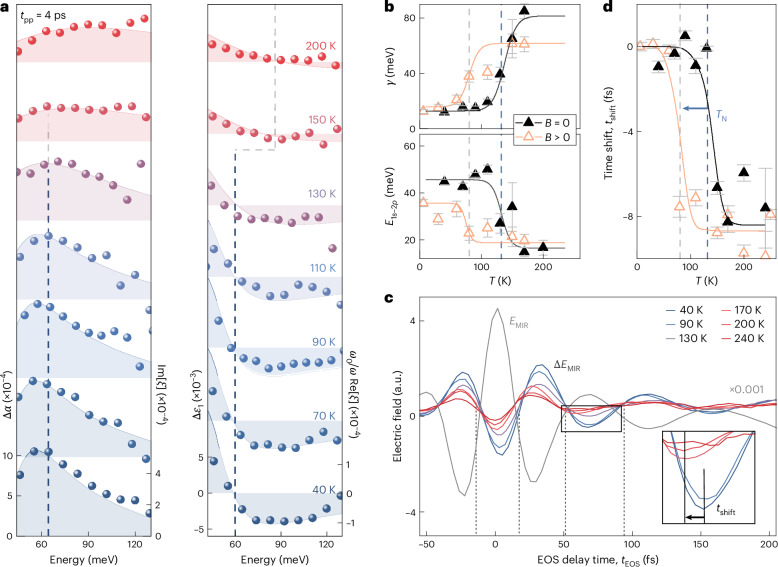
Controlling the effective dimensionality of excitons by the magnetic phase transition. **a**, Pump-induced changes in the MIR absorption (Δ*α*) and the real part of the dielectric function (Δ*ε*_1_) as a function of photon energy at a pump–probe delay time *t*_pp_ = 4 ps for different temperatures *T* (colour coded; pump fluence, *Φ*_NIR_ = 300 μJ cm^–2^). Curves are vertically offset for clarity. The spheres and the shaded areas represent the experimental and theoretical data, respectively. All right axes show the function *ξ* defined in the Methods. The vertical dashed lines track the maximum of Δ*α* and the corresponding zero crossing of Δ*ε*_1_, revealing an abrupt jump close to *T*_N_. **b**, Theoretically calculated dephasing constant *γ* and transition energy *E*_1*s*−2*p*_ as a function of *T*. The symbols represent the values derived from the fit to the experimental data shown in **a**. The error bars of *γ* and *E*_1*s*−2*p*_ are derived using the covariance matrix of the nonlinear fit to the experimental data shown in **a**. **c**, Electric field of the MIR probe transient *E*_MIR_ (scaled by a factor of 10^−3^ for comparison) as a function of the electro-optic sampling (EOS) time *t*_EOS_. The simultaneously acquired pump-induced changes to the electric field Δ*E*_MIR_ are depicted for different temperatures. Inset: close-up view of the data marked by the rectangle. For decreasing temperature, the minimum of Δ*E*_MIR_ shifts gradually by the time *t*_shift_ (arrow), as shown in **d**. Analogous analyses for other oscillation half-cycles are shown in Supplementary Fig. 2. **d**, Experimental (black symbols) and theoretical (black line) time shift *t*_shift_ of the half-cycle marked by the rectangle in **c** as a function of *T* (compared with the timing at *T* = 40 K) without an external magnetic field. *T*_N_ is marked by a vertical dashed blue line. An applied magnetic field of 200 mT along the *b* axis induces an intermediate FM phase at *T*′ causing the jump in *t*_shift_ in both experiment and theory (orange symbols and line, respectively) to occur at only ~80 K (arrow and grey dashed line; Supplementary Fig. 3). The error bars represent the 95% confidence interval of the fitting procedure used to determine the extrema of the waveforms.

Because the excitonic fine structure maps electron–hole interactions, it represents a unique tool to study the impact of magnetic order on Coulomb correlations. We start by following how the intraexcitonic 1*s*–2*p*_*b*_ resonance changes from the AFM to PM phase by tuning the lattice temperature. Figure 3a shows the measured MIR response functions (spheres). The pump–probe delay time is kept large enough (*t*_pp_ = 4 ps) for the photoexcited electron–hole pairs to form excitons and reach a quasi-equilibrium distribution. This also suppresses the inhomogeneous broadening of the 1*s*–2*p* transition by the contribution of higher-lying 1*s*-exciton–polariton states (Methods). For *T* = 40 K, the 1*s*–2*p*_*b*_ resonance gives rise to the same fingerprints in Δ*α* and Δ*ε*_1_ at an energy of 62 meV as those shown in Fig. 2a. On increasing the temperature up to *T*_N_ = 132 K, the resonance energy remains nearly unchanged (Fig. 3a, dashed vertical line), whereas the linewidth broadens. By contrast, at the transition to the PM phase^37^, the linewidth increases abruptly and the apparent resonance energy shifts.

A rigorous theory–experiment comparison allows us to quantitatively link the spectral response to the temperature-dependent microscopic 1*s*–2*p* transition energy *E*_1*s*−2*p*_ and the dephasing constant *γ*, which determines the spectral broadening (Fig. 3b and Methods). Because we demand our many-body theory to simultaneously reproduce Δ*α* and Δ*ε*_1_, instead of simply analysing only one of these quantities, the error bars for *E*_1*s*−2*p*_ remain on the few-millielectronvolt level for all temperatures (Fig. 3b, error bars). Remarkably, both *E*_1*s*−2*p*_ and *γ* switch abruptly at around *T* = *T*_N_, explaining the sudden changes in the spectral response. Whereas *γ* jumps by more than a factor of 6, the polariton-corrected value of *E*_1*s*−2*p*_ exhibits a crossover from tightly bound (50 meV) to weakly bound (15 meV) excitons.

**Extended Data Fig. 2:**
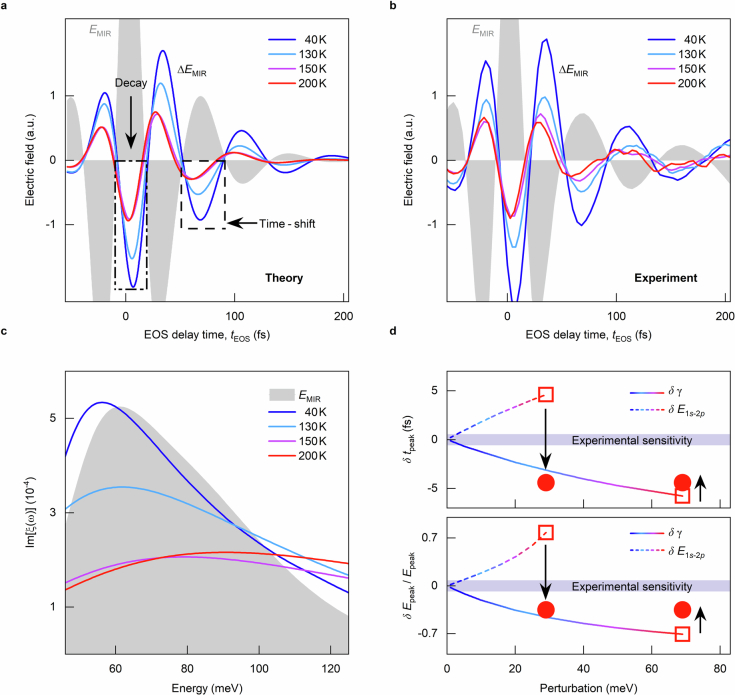
Connection between measured temporal transients and excitonic response. **a**, Computed versus **b**, measured pump-induced changes of the MIR electric field transients, ∆*E*_MIR_ (*t*_EOS_), as a function of the electro-optic sampling time *t*_EOS_, are shown at four representative temperatures. The dotted box marks the time window of interest for the decay analysis in Fig. 4 while the dashed box corresponds to the time-shift analysis of Fig. 3. **c**, Computed MIR spectral response, Im[ξ(ω)], as a function of the photon energy corresponding to the electric field transients shown in **a**. **d**, Change in the temporal position, δ*t*_peak_ (top), of the first half-cycle of ∆*E*_MIR_ (*t*_EOS_) and change in the corresponding relative peak amplitude, δ*E*_peak_/*E*_peak_ (bottom), as a function of the perturbation of the broadening parameter, δγ (solid lines), or the 1*s*–2*p* transition energy, δ*E*_1*s*-2*p*_ (dashed lines). Zero perturbation corresponds to the response at *T* = 40 K (see **c**, dark blue line). The squares indicate δ*t*_peak_ and δ*E*_peak_/*E*_peak_ computed for the cases where only δγ and δ*E*_1*s*-2*p*_ are individually varied to values corresponding to the response function at *T* = 200 K (see **c**, red curve). Filled circles mark the result of the simultaneous perturbation of δγ and δ*E*_1*s*–2*p*_ by the same amount given by the position of the data points along the horizontal axis. Arrows indicate the changes between individual and simultaneous perturbations. Shaded horizontal squares assign the detection threshold set by the experimental sensitivity.

**Extended Data Fig. 3:**
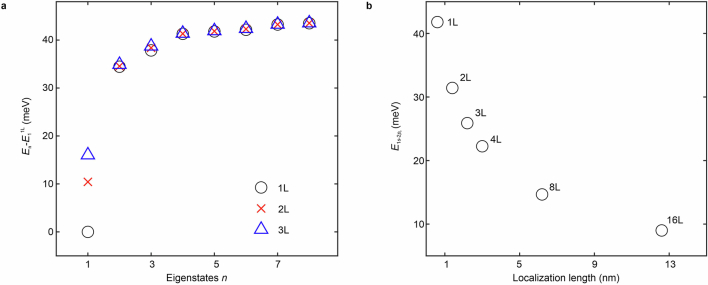
Localization dependence of the internal excitonic structure. **a**, Calculated exciton series for different electron–hole wavefunction localization across 1, 2 or 3 layers. Energies are relative to the ground state energy of the single-layer solution. **b**, Transition energy between the 1*s* ground state and the 2*p*_b_ state (5^th^ eigenstate) as a function of the electron–hole wavefunction localization along the crystal *c*-axis. To relate the localization length to the number of layers, we consider a layer thickness of 6 Å as well as an interlayer separation of 2 Å, consistent with relaxed lattice geometries from first-principles calculations^26,27^.

**Extended Data Fig. 4:**
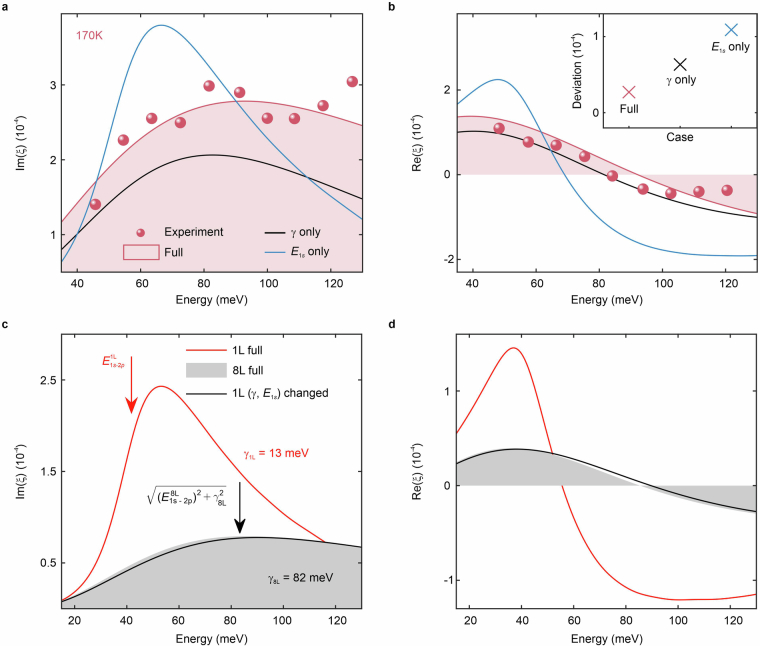
Illustration of the impact of delocalization on the MIR response and spectral analysis to disentangle shift and broadening effects. **a,b**, A maximum likelihood estimate to the measured imaginary (**a**) and real (**b**) part of the MIR response (spheres) is obtained using the full calculation (red shaded area) including exciton delocalization and broadening effects and result in a comparable value for *t*_shift_. Optimizing only the broadening γ (black line) or only the *E*_1*s*_ energy (blue line) to obtain *t*_shift_ results in substantial spectral deviations as quantified by the root mean square error (inset in **b**). Full spectral information is necessary to disentangle shift and broadening effects. Results are shown for a temperature of *T* = 170 K and without an external magnetic field. **c,d**, Imaginary (**c**) and real (**d**) part of the MIR susceptibility for localization of the exciton wavefunction to a single layer (1 L, red line) and to eight layers (8 L, shaded area), respectively. The polarization of the MIR probe is chosen along the *b* direction of the CrSBr crystal. At low temperatures, the absorption peak is well described by the 1*s*-2*p* resonance energy (red arrow), while it gets strongly renormalized (black arrow) with increasing the broadening γ induced by the delocalization of excitons. The delocalized response can be accurately approximated by a localized calculation together with an 1*s*-exciton energy shifted by the energy reduction caused by the delocalization (black line).

These features are consistent with a phase transition from 1D to three-dimensional excitons. Thermal fluctuations relax the constraints induced by the magnetic order, which delocalizes the exciton wavefunction across adjacent layers. This increase in the localization length results in a monotonic reduction in the 1*s* energy *E*_1*s*_, whereas the other states remain largely unaltered (Extended Data Fig. 3). Overall, this leads to a reduction in the exciton binding energy and an increase in the scattering rates and, thus, dephasing *γ* (Methods). This trend shifts *E*_1*s*−2*p*_ defining the position of the MIR absorption peak (Fig. 3 and Extended Data Fig. 2). As shown in Fig. 2a, the 1D exciton yields a distinct asymmetric resonance close to *E*_1*s*−2*p*_. By contrast, three-dimensional excitons feature a broad spectrum (Fig. 3a) in which the very large *γ* renormalizes the spectrum to show a shallow resonance close to the energy corresponding to *γ* (Extended Data Fig. 4).

The change in the spectral response functions leaves unique fingerprints even in the raw time-domain data (Fig. 3c). For all investigated temperatures, the pump-induced change in the MIR electric field Δ*E*_MIR_ is phase-shifted by π with respect to the reference electric field *E*_MIR_, indicating an attenuation of the transmitted MIR field through absorption by intraexcitonic transitions. For *T* < *T*_N_, the well-defined 1*s*–2*p*_*b*_ resonance additionally manifests as pronounced retarded oscillations persisting up to *t*_EOS_ = 200 fs. These signatures are absent in the PM phase (*T* > *T*_N_), owing to the weak and massively broadened exciton response, as confirmed by our theory (Methods). The disappearance of a narrow spectral feature in the response function considerably shifts the extrema of Δ*E*_MIR_ for all trailing half-cycles. Figure 3c (inset) illustrates this time shift *t*_shift_ for one half-cycle (rectangle). The temperature dependence of *t*_shift_ (*T*) with respect to *t*_shift_ (*T* = 40 K) (Fig. 3d, black triangles) exhibits an abrupt jump at around *T*_N_ = 132 K; the other half-cycles yield a similar discontinuity in *t*_shift_ (Supplementary Fig. 2). Our many-body calculations fully capture the observed behaviour (Fig. 3d, solid curve). Although full spectral information was required to disentangle the effects of the renormalization and broadening of excitonic energy, the experimentally observed *t*_shift_ effectively reveals the essence of the temperature-induced changes in the internal structure of excitons.

To confirm that the observed drastic changes in the exciton fine structure result from the spin order and not from thermal effects, we induce an intermediate FM phase close to the AFM–PM phase transition using a static magnetic field of *B*_stat_ = 200 mT applied along the easy axis^36^ (*b* axis). By repeating the same experiment–theory comparisons as those shown in Fig. 3a,c under magnetic bias (Supplementary Fig. 3), we observe a similarly abrupt jump in *t*_shift_ with temperature (Fig. 3d, orange triangles). In stark contrast to the case without an external magnetic field, this step already occurs at a temperature that is reduced by about 50 K. This new transition temperature *T*′ < *T*_N_ indicates that *B*_stat_ induces a change in the magnetic structure and, more precisely, forces an interlayer FM ordering much below the Néel temperature. This scenario is in good agreement with the field-induced AFM–FM transition observed in recent magnetometry studies on CrSBr (refs. ^41,42^). Therefore, the external magnetic field results in the breakdown of the magnetic-order-induced quantum confinement of excitons into a single monolayer of CrSBr and delocalizes them even below *T*_N_. Our results introduce the magnetic field as a readily tunable parameter to custom-tailor the internal exciton structure in magnetic vdW materials, which has not been possible in any other two-dimensional material so far, to the best of our knowledge.

## Impact of magnetic order on exciton decay dynamics

**Fig. 4:**
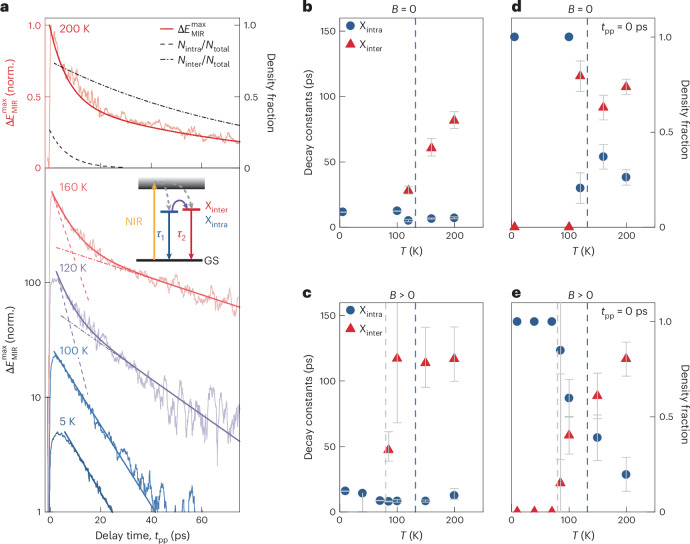
Ultrafast recombination dynamics and coexistence of different excitonic species. **a**, Maximum of the pump-induced change in the MIR electric field $\Delta {E}_{{\rm{MIR}}}^{\max }$ as a function of the pump–probe delay time *t*_pp_ for a fixed electro-optic sampling time *t*_EOS_ (light-coloured lines; *Φ*_NIR_ = 225 μJ cm^–2^) recorded at different temperatures *T*. The data on a logarithmic scale are vertically offset for clarity. The darker-coloured lines represent mono- and biexponential decay fits. For clarity, a ten-point (thirty-point) moving average was applied for delay times smaller (larger) than 20 ps. The dashed lines indicate the isolated fast- and slow-decay components of the fit and highlight the deviation from monoexponential decay at elevated temperatures. Top: density fraction of intralayer and interlayer excitons as a function of *t*_pp_ at *T* = 200 K (dashed lines). **b**, Decay times of intralayer (*τ*_1_; blue circles) and interlayer (*τ*_2_; red triangles) excitons as a function of *T* obtained from the fit in **a**. *τ*_2_ rises close to *T*_N_ (dashed line). **c**, *τ*_1_ and *τ*_2_ as a function of *T* with an applied magnetic bias (*B*_stat_ = 200 mT) along the *b* axis, obtained from the fit in Extended Data Fig. 6. The slower decay component (*τ*_2_) emerges at *T*′ ≈ 80 K (grey dashed line). **d**,**e**, Density fraction of intra- and interlayer excitons as a function of *T* without (**d**) and with (**e**) an applied magnetic field. The extraction procedure is described in Supplementary Section 4. The error bars represent the 95% confidence interval of the fitting routine. For the temperatures featuring only the decay component *τ*_1_ in **b** and **c** and no error bars in **d** and **e**, a monoexponential decay was sufficient to describe the experimental data reasonably well. Panels **b**–**e** share the same colour code. The inset in **a** shows the level scheme of interlayer-like (X_inter_) and intralayer-like (X_intra_) excitons relative to the continuum (shaded region) and the ground state (GS). Upon excitation by the NIR pulse (yellow arrow), both exciton species can form (dashed arrows) and decay with rates *τ*_1_ and *τ*_2_. Elevated lattice temperatures facilitate the population of X_inter_ (purple arrow), resulting in a decrease (increase) in the fraction of the short-lived (long-lived) exciton species.

**Extended Data Fig. 5:**
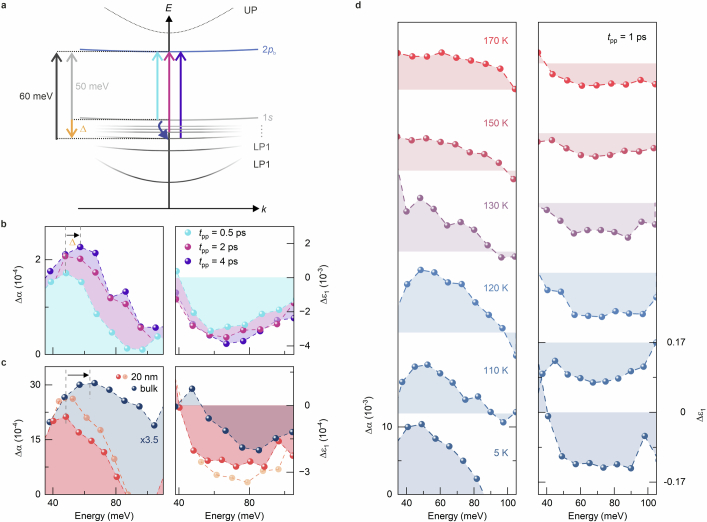
Polaritons renormalize the resonance energy. **a**, Schematic illustration of the dispersion relation of exciton-polaritons in bulk CrSBr. Due to strong self-hybridization effects, the 1*s* state (solid grey line) splits into multiple lower polariton (LP, grey-scale intensity illustrates the exciton fraction) and upper polariton (UP, dotted grey line) states. The 2*p*_b_ state is indicated by the blue line. In the low fluence regime and at early delays, the MIR pulse probes cavity polariton states close to the bare 1*s*-2*p*_b_ transition (50 meV, light blue arrow). The subsequent relaxation of the polaritons towards energetically lower-lying states (curved arrow) results in a blueshift (pink and purple arrows) of the 1*s*-2*p*_b_ transition by ∆ (yellow arrow). Energy separations are not drawn to scale. **b**, Pump-induced change of the MIR absorption (∆α) and the real part of the dielectric function (Δε_1_) as a function of the photon energy for different pump-probe delay times *t*_pp_ (pump fluence Φ_NIR_ = 75 μJ/cm^2^). The arrow indicates a blueshift of the maximum in ∆α by ∆ for later delay times. **c**, ∆α and Δε_1_ for a 20-nm-thin CrSBr flake (*t*_pp_ = 1 ps: red symbols, red shading; *t*_pp_ = 0.1 ps: orange symbols, no shading) as well as a bulk crystal (*t*_pp_ = 1 ps: blue symbols and shading). The arrow indicates a blueshift of the maximum in ∆α for the bulk sample due to polaritonic corrections. **d**, Temperature-dependent internal structure of excitons in 20-nm-thin CrSBr. ∆α and Δε_1_ as a function of the photon energy at a delay time of *t*_pp_ = 1 ps at different temperatures (Φ_NIR_ = 600 μJ/cm^2^).

The influence of spin order on the effective exciton confinement should also be imprinted on the ultrafast dynamics of these quasiparticles. To investigate this connection, we record the maximum of the pump-induced change in the MIR field transient, $\Delta {E}_{{\rm{MIR}}}^{\max }$, as a function of the pump–probe delay time *t*_pp_, for temperatures below and above *T*_N_ (Fig. 4a). The evolution of $\Delta {E}_{{\rm{MIR}}}^{\max }$ closely follows the exciton density; it abruptly increases during photoexcitation before it decays on a picosecond scale. Whereas a subtle change in the onset as a function of temperature reflects polaritonic corrections^43^ (Methods and Extended Data Fig. 5), we mainly focus on the more relevant decay dynamics. In the AFM phase, the relaxation follows a single exponential function with a decay constant of *τ*_1_ ≈ 13 ps representing the recombination of quasi-1D excitons, where electrons and holes are located in the same layer (intralayer excitons).

Close to the magnetic phase transition, however, an additional slower decay component with a lifetime of *τ*_2_ > 60 ps emerges as the fast decay persists (Fig. 4b). We assign the short-lived decay to quasi-1D intralayer excitons for which a strong electron–hole spatial overlap yields a short lifetime (*τ*_1_), whereas the weak overlap among delocalized interlayer excitons implies increased *τ*_2_ ≫ *τ*_1_. As shown in Supplementary Section 4, we can accurately convert the relative amplitudes of the fast and slow components to the decay dynamics of the actual inter- and intralayer exciton fractions (Fig. 4a (top), broken lines) or track them as functions of temperature (Fig. 4d). Whereas only intralayer excitons exist for *T* < *T*_N_, both inter- and intralayer excitons occur above *T*_N_. This behaviour unveils a key aspect of the excitons at play: the phase transition into the PM phase does not change the dimensionality of all excitons at once, but rather enables a coexistence of different species. When the AFM order is quenched, spin restrictions on interlayer tunnelling are lifted, and some of the electrons and holes can move between adjacent layers. Consequently, electron–hole pairs can form both inter- and intralayer excitons as the thermodynamic equilibrium is approached in the level system indicated in Fig. 4a (inset).

**Extended Data Fig. 6:**
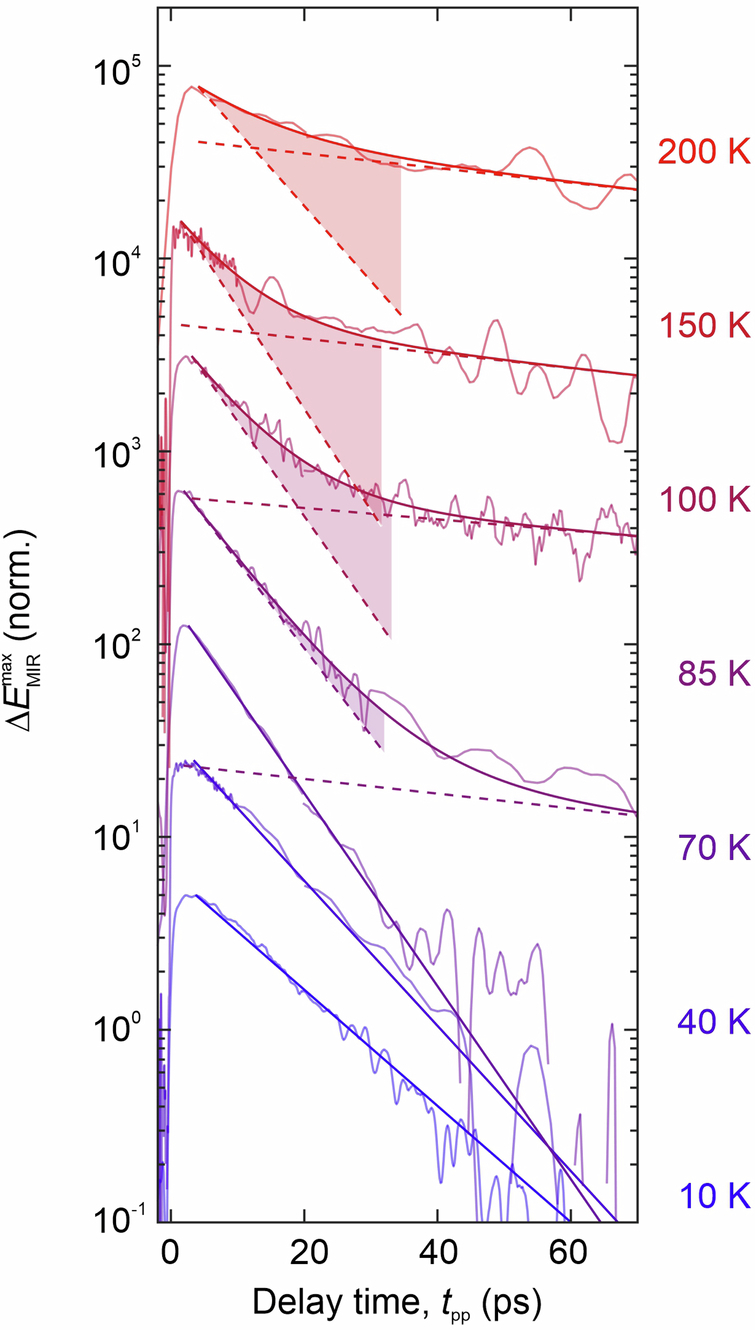
Ultrafast recombination dynamics with an applied magnetic field. Maximum of the pump-induced change of the MIR electric field $\Delta {E}_{{\rm{MIR}}}^{{{\max }}}$ as a function of the pump-probe delay time *t*_pp_ for a fixed EOS time *t*_EOS_ (light-coloured lines, pump fluence Φ_NIR_ = 300 µJ/cm^2^) recorded at different temperatures (colour code) with an applied magnetic field of *B* = 200 mT along the *b* axis. The data on a logarithmic scale are vertically offset for clarity. The darker-coloured lines represent fits to the exciton dynamics with biexponential decays. For clarity, a two (five) point moving average was applied for delay times smaller (larger) than 20 ps. For the fitting procedure the raw data was used. The dashed lines show the isolated fast and slow decay components of the fit and the shaded regions highlight the deviation from monoexponential decay dynamics. The extracted parameters are summarized in Fig. 4c,e.

Finally, we put this scenario to its ultimate test, by applying a magnetic bias of *B*_stat_ = 200 mT (Fig. 3b,d), which can enforce an AFM–FM phase transition below *T*_N_. Intriguingly, the slow-decay component now occurs for temperatures as low as *T*′ = 80 K (Fig. 4c and Extended Data Fig. 6), where also the density of interlayer excitons sets on (Fig. 4e). These findings demonstrate that the magnetic order not only controls the effective dimensionality and fine structure of excitons in CrSBr, but also the coexistence of different exciton species.

## Discussion

We have directly resolved the anisotropic fine structure of quasi-1D excitons by intraexcitonic Rydberg spectroscopy. Our quantitative experiment–theory comparisons of the exciton binding energy reveal how the band structure and Coulombic anisotropy lift the degeneracy of the 2*p* states and induce a strong direction-dependent oscillator strength. In the AFM state, the excitons are layer localized even in the bulk material, resulting in quasi-1D excitons. Forcing a phase transition, either by thermal heating above the Néel temperature or by applying a weak magnetic field, facilitates electronic hopping between neighbouring layers. This scenario relaxes the quantum confinement, reducing the electron–hole binding energy and dramatically enhancing the phase space for scattering. Our results, thus, introduce magnetic order as a critical tuning knob for excitonic correlations and highlight a pathway to switch exciton properties in CrSBr in future spintronic applications. As a next step, the strong interaction between excitons and magnetic order could be further tuned by proximity effects^44–46^. In this context, it would be of particular interest to study few-layer and monolayer samples^47^ with larger exciton binding energies. Moreover, strong light–matter coupling renders CrSBr a prime candidate for creating exciton–polaritons^27,48^ with strongly and simultaneously coupled electronic, magnetic and photonic degrees of freedom. In particular, our magnetic control of the internal structure of excitons could open a unique playground for interfacing macroscopic wavefunctions of exciton–polariton condensates with spintronics—a spectacular vision for novel solid-state quantum technologies.

## Methods

### Time-resolved NIR pump–MIR probe spectroscopy

A schematic of the experimental setup is shown in Extended Data Fig. 1a. Our home-built Ti:sapphire laser amplifier (repetition rate, 400 kHz) delivers ultrashort NIR pulses with a duration of 20 fs (full-width at half-maximum). The NIR pulses are split into pump, probe and gate branches. A half-wave plate in the pump branch was installed to excite excitons along the *b* direction and to probe either along the *a* or *b* direction of the CrSBr crystal. Optical rectification of ultrashort NIR pulses in a 10-µm-thick GaSe crystal is used to generate phase-locked MIR probe pulses. After a variable delay time *t*_pp_, an MIR probe pulse propagates through the photoexcited sample. The transmitted MIR waveforms are superimposed with ultrashort gate pulses onto a second 10-μm-thick GaSe electro-optic crystal to record the electric field *E*_MIR_ as a function of the electro-optic sampling time *t*_EOS_ (Extended Data Fig. 1b). The MIR probe pulses are centred at a frequency of 17 THz with a full-width at half-maximum of 15 THz and an approximately flat spectral phase between 15 and 40 THz (Extended Data Fig. 1c). The changes to the electric field, Δ*E*_MIR_(*t*_EOS_), transmitted through the excited and unexcited sample are directly resolved in absolute amplitude and phase by serial lock-in detection as a function of *t*_EOS_. We use a transfer matrix formalism technique to extract the full complex-valued dielectric response function, characterized by Δ*α* and Δ*ε*_1_ (refs. ^6,32,40^). These spectroscopic data are sensitive to the total population of bound and unbound electron–hole pairs, irrespective of interband selection rules and can, thus, address optically dark and bright excitonic states.

### Theoretical analysis

To describe the MIR response, we utilize a full many-body theory as presented in other work^30,34^. Exciton states and energies that determine the internal structure and dipole moments of intraexcitonic transitions are obtained by solving an anisotropic Wannier equation. We obtain the necessary material parameters from ab initio density functional theory band structure calculations including many-body corrections within the *GW* approximation^26^. The dielectric screening of the Coulomb interaction is described via an anisotropic dielectric function derived from Poisson’s equation^49^ for a CrSBr monolayer embedded in bulk CrSBr. Our computations incorporate spatial non-locality via the in-plane momentum dependence of the dielectric function, resulting from single-layer-localized electronic states in the AFM state^25,26^ and multilayer-extended states in the PM state.

Since the MIR wavelength is much longer than the sample thickness *L*, the MIR field propagates through an effectively thin two-dimensional layer whose intrinsic optical properties are described by an effective two-dimensional susceptibility *χ*_2D_(*ω*) (in units of metres) as a function of the angular frequency of the MIR field. We compute the full many-body *χ*_2D_(*ω*) via the steps outlined above. Following the discussion in refs. ^28,34^, it is useful to introduce a dimensionless, scaled susceptibility ${\rm{\xi }}\left({\rm{\omega }}\right)=\frac{{\rm{\omega }}}{2c}{\chi }_{2{\rm{D}}}\left({\rm{\omega }}\right)$ containing the speed of light *c* in a vacuum. This response function *ξ*(*ω*) connects computations directly with the measured transmission amplitude *T*(*ω*) and linear absorption *α*(*ω*) via $T\left({\rm{\omega }}\right)=\frac{1}{1-i{\rm{\xi }}\left({\rm{\omega }}\right)}$ and ${\rm{\alpha }}\left({\rm{\omega }}\right)=\frac{2{\rm{Im}}\left[{\rm{\xi }}\left({\rm{\omega }}\right)\right]}{{\left|1-i{\rm{\xi }}\left({\rm{\omega }}\right)\right|}^{2}}$ that become *T*(*ω*) → 1 + i*ξ*(*ω*) and *α*(*ω*) → 2Im[*ξ*(*ω*)] for the small *|ξ*(*ω*)| ≪ 1 studied here. Additionally, the pump-induced dielectric change becomes $\Delta {{{\varepsilon }}}_{1}=\mathrm{Re}\left[\frac{2c}{{{\omega }}L}{\rm{\xi }}\left({{\omega }}\right)\right]$. With the help of the measured MIR probe spectrum (Extended Data Fig. 1), we predict the pump-induced changes to the transmitted electric field as Δ*E*(*ω*) = i*ξ*(*ω*)*E*_MIR_(*ω*) to determine Δ*E*(*t*_EOS_) and the time shift *t*_shift_ of each half-cycle in the time domain. Extended Data Fig. 2a,b compares the measured versus computed Δ*E*(*t*_EOS_), respectively, for four representative temperatures. In all these cases, theory reproduces experimental details with great precision.

### Precision of delay-based measurement in detecting excitonic features

Since our theory rigorously connects the temporal traces, Δ*E*(*t*_EOS_), to the computed excitonic MIR responses, *ξ*(*ω*), we can quantitively determine how accurately our temporal-shift measurements (Fig. 3) can track the changes in the excitonic dephasing *γ* or the 1*s*–2*p* transition energy *E*_1*s*–2*p*_; Extended Data Fig. 2c shows Im[*ξ*(*ω*)] corresponding to the four temporal traces in Extended Data Fig. 2a. Among these cases, *γ* changes by δ*γ* = 70 meV and *E*_1*s*–2*p*_ by δ*E*_1*s*–2*p*_ = 35 meV as the temperature rises from 40 K to 200 K.

To precisely quantify the minimum experimentally detectable excitonic changes, we start from *ξ*(*ω*) at 40 K, separately vary δ*γ* and δ*E*_1*s*–2*p*_ computationally and determine the resultant changes in the timing of the peak field (δ*t*_peak_) and the relative peak field strength (δ*E*_peak_/*E*_peak_) at the electric field extrema (Extended Data Fig. 2a, dashed rectangle). These values are compared with the experimental temporal (Δ*t*_res_) and peak-field-strength (Δ*E*_peak_) precision values determined from four consecutive measurements performed at a temperature of 40 K. The standard deviation of these measurements yields Δ*t*_res_ = 0.5 fs and Δ*E*_peak_/*E*_peak_ = 5%. Specifically, Extended Data Fig. 2d shows the changes in δ*t*_peak_ (top) and δ*E*_peak_/*E*_peak_ (bottom) as a function of perturbations δ*γ* (solid lines) or δ*E*_1*s*–2*p*_ (dashed lines). We conclude that variations in δ*γ* and δ*E*_1*s*–2*p*_ as small as 5 meV already produce changes above the experimental detection thresholds of Δ*t*_res_ and Δ*E*_peak_/*E*_peak_ (Extended Data Fig. 2d, shaded area). At 200 K, separate changes of δ*γ* = 70 meV or δ*E*_1*s*–2*p*_ = 35 meV alone yield the results indicated by the open squares. In the actual measurements, simultaneous changes in δ*γ* and δ*E*_1*s*–2*p*_ are connected (solid circles in Extended Data Fig. 2d); the arrows indicate the response changes between the individual (open squares) and simultaneous (solid circles) variations in δ*γ* and δ*E*_1*s*–2*p*_.

These cases considerably exceed the detection thresholds, corroborating that our measurements and temporal analysis can sensitively assign the key excitonic features *γ*, *E*_1*s*–2*p*_ and *ξ*(*ω*) reported in the main text. Although *t*_shift_ depends on both δ*γ* and δ*E*_1*s*–2*p*_, it alone cannot assign an exact value for them because multiple combinations of δ*γ* and δ*E*_1*s*–2*p*_ produce the same *t*_shift_ (Extended Data Fig. 2d). However, our combined spectral–*t*_shift_ analysis uniquely assigns (*γ*, *E*_1*s*−2*p*_) from the experiments, as confirmed in Extended Data Fig. 2.

### Impact of exciton localization and scattering on the internal exciton structure

To analyse the temperature-dependent MIR response, we first identify the connection of many-body and measurable quantities. At low temperatures, the AFM order prevents interlayer hybridization and localizes the exciton wavefunction within a single layer^25,26^. Thermal fluctuations relax the constraints induced by the magnetic order, which, in turn, allows the exciton wavefunction to spread across adjacent layers. This spreading reduces both electron–hole Coulomb interaction and exciton binding energy. The underlying localization length scale *d* modifies the unscreened Coulomb potential $U(|{\bf{q}}|)=\frac{{e}^{2}}{2{\varepsilon }_{0}q}F(q)$ through a form factor^50^ $F\left(q\right)=\frac{2}{\uppi }\arctan \left(\frac{\uppi }{{qd}}\right)$. Extended Data Fig. 3a shows the exciton series for varying extensions of the wavefunction over adjacent layers. Increasing the localization length results in a monotonic reduction in energy *E*_1*s*_, whereas the other states remain essentially unchanged. This trend shifts the *E*_1*s*−2*p*_ energy defining the position of the MIR absorption peak (Extended Data Fig. 2 and Fig. 3). Figure 3b displays the values of *E*_1*s*−2*p*_ = 46 meV below and *E*_1*s*−2*p*_ = 16 meV above *T*_N_, respectively. On the basis of Extended Data Fig. 3b, this change in *E*_1*s*−2*p*_ corresponds to a transition from a single monolayer (1 ML) to eight monolayers (8 ML) as *T*_N_ is crossed. The spreading across 8 ML also increases the effective dimensionality of electrons and holes as the confinement is relaxed, enabling new transition and scattering possibilities across the layers. The increased scattering induces an elevated dephasing *γ*. As shown in Fig. 3b, *γ* grows by more than a factor of six when the system undergoes the Néel transition, which corroborates a substantial increase in the exciton’s dimensionality.

To illustrate the effects of delocalization and broadening, Extended Data Fig. 4c,d compares the MIR response at a low temperature (*T* = 40 K; localization to 1 ML) to a delocalized case (shaded area; spreading across 8 ML) with substantially enhanced *γ*. The corresponding 1*s*–2*p* transition energies are ${E}_{1s-2p}^{1{\rm{L}}}=42\,{\rm{meV}}$ for the 1 ML case and ${E}_{1s-2p}^{8{\rm{L}}}=15\,{\rm{meV}}$ for the 8 ML case. We observe that the peak in the MIR absorption follows the 1*s*–2*p* energy (red arrow) only for the 1 ML case, whereas the 8 ML peak shifts to higher energies contrary to the reduced *E*_1*s*−2*p*_. This seemingly counterintuitive trend is explained by the considerably increased dephasing (from *γ*_1L_ = 13 meV to *γ*_8L_ = 82 meV) that renormalizes^51^ the observed absorption peak to an energy ${\widetilde{E}}_{1s-2p}=\sqrt{{\left({E}_{1s-2p}\right)}^{2}+{{\rm{\gamma }}}^{2}}$ (Extended Data Fig. 4c, black arrow), and causes a substantial blueshift in the MIR absorption peak.

### Accurate parameterization of the Néel transition

As shown above, localization yields a strong *d* dependence for *γ* and, in particular, *E*_1*s*_ from the entire Rydberg series. This identifies (*γ*, *E*_1*s*−2*p*_) as the minimum set of *d*-dependent many-body quantities; we use the experimentally measured intraexcitonic energy separation *E*_1*s*−2*p*_ to single out the relevant Coulomb-induced many-body effects from bandgap renormalization effects.

To test whether (*γ*, *E*_1*s*−2*p*_) parameterizes the Néel transition, we compare a full 8 ML computation including all many-body changes with a 1 ML computation that is adjusted to match the 8 ML one by changing only (*γ*, *E*_1*s*−2*p*_). Specifically, Extended Data Fig. 4a,b shows the scaled susceptibility, *ξ*(*ω*), for the full 1 ML (red line, *γ* = 13 meV, *E*_1*s*−2*p*_ = 42 meV), full 8 ML (shaded area, *γ* = 82 meV, *E*_1*s*−2*p*_ = 15 meV) and adjusted 1 ML (black line, *γ* = 82 meV, *E*_1*s*−2*p*_ = 15 meV) cases. The change from the full 1 ML to full 8 ML is dramatic, as expected when the system undergoes a Néel transition. However, the adjusted 1 ML computation accurately predicts the 8 ML result only when the (*γ*, *E*_1*s*−2*p*_) pair is changed in an otherwise consistent computation.

Thus, we have verified that the experimentally studied dimensionality control (via the Néel transition) can be parameterized via (*γ*, *E*_1*s*−2*p*_) alone. This allows us to similarly add the *E*_1*s*_ polariton shift to *E*_1*s*−2*p*_ (see the section below) and strongly reduce the numerical effort because the many-body details need to be computed only once, whereas a (*γ*, *E*_1*s*−2*p*_) pair accurately describes the dimension-dependent effects. We have applied this scheme in Figs. 2b and 3 and Extended Data Fig. 4 to accurately include a polariton correction of *Δ* = 10 meV to *E*_1*s*−2*p*_.

### Full spectral analysis

To map the temperature dependence of the (*γ*, *E*_1*s*−2*p*_) parameters, we carefully assign the maximum likelihood (*γ*, *E*_1*s*−2*p*_) corresponding to each measurement. We simply convert the measured MIR transmission coefficient *T*(*ω*) to the scaled susceptibility ${\rm{\xi }}\left({\rm{\omega }}\right)={\rm{i}}\frac{1-T\left({\rm{\omega }}\right)}{T\left({\rm{\omega }}\right)}$. This allows us to compare the experimental *ξ*_exp_(*ω*) and computed *ξ*(*ω*) values, as well as the absorptive (Im[*ξ*_exp_(*ω*)]) and dispersive (Re[*ξ*_exp_(*ω*)]) responses, in the same absolute units to have a reliable assignment of maximum likelihood. Specifically, we minimize the least-mean square deviation, Mean[|*ξ*(*ω*) – *ξ*_exp_(*ω*)|^2^], as a function of (*γ*, *E*_1*s*−2*p*_). Extended Data Fig. 4a,b shows the maximum-likelihood solution (red line) to the measured response (spheres) at *T* = 170 K without an external magnetic field. The calculated time shift *t*_shift_ of the time transient is comparable with the experimental estimate (*t*_shift_ = –8 fs, third half-cycle). As discussed above, the same *t*_shift_ can be obtained by changing only *γ* or only *E*_1*s*−2*p*_ compared with the 170 K experiment. Adjusting either *γ* (black line) or *E*_1*s*−2*p*_ (blue line) individually to achieve *t*_shift_ of –8 fs results in notable spectral deviations (Extended Data Fig. 4b, inset). This confirms that using the full spectral information is necessary to accurately determine a unique (*γ*, *E*_1*s*−2*p*_) pair from the *t*_shift_ measurements, as demonstrated in the main text.

### Phase transition model

The temperature-dependent maximum-likelihood (*γ*, *E*_1*s*−2*p*_) parameters are shown as symbols in Fig. 3b. Clearly, both parameters exhibit a strong change above *T*_N_ (marked as the dashed blue line), and the magnetic field shifts the transition temperature to a smaller value (dashed grey line). On the basis of these data, (*γ*, *E*_1*s*−2*p*_) closely follows the empirical phase transition model below:

$$\begin{array}{c}\gamma (T\,)=\frac{{\gamma }_{\infty }-{\gamma }_{0}}{2}\,\tanh\left(\frac{T-{T}_{\gamma }}{\Delta {T}_{\gamma }}\right)+\frac{{\gamma }_{\infty }+{\gamma }_{0}}{2};\\ {E}_{1s-2p}(T\,)=\frac{{E}_{\infty }-{E}_{0}}{2}\,\tanh\left(\frac{T-{T}_{\varDelta }}{\Delta {T}_{E}}\right)+\frac{{E}_{\infty }+{E}_{0}}{2}\end{array}$$

with fitting parameters for the transition temperature (*T*_*γ*_ and *T*_*Δ*_), transition rate (Δ*T*_*γ*_ and Δ*T*_*E*_), and low (*γ*_0_ and *E*_0_) and high (*γ*_∞_ and *E*_∞_) temperature limits. Without an external magnetic field, we find *T*_*γ*_ = 137.5 K, *T*_*Δ*_ = 132.5 K, Δ*T*_*γ*_ = 18.9 K, Δ*T*_*E*_ = 14.8 K, *γ*_0_ = 12.8 meV, *E*_0_ = 45.7 meV, *γ*_∞_ = 81.6 meV and *E*_∞_ = 16.5 meV; and with a magnetic field, *T*_*γ*_ = 80 K, *T*_*Δ*_ = 75 K, Δ*T*_*γ*_ = 16.2 K, Δ*T*_*E*_ = 9.1 K, *γ*_0_ = 15.8 meV, *E*_0_ = 35.7 meV, *γ*_∞_ = 61.7 meV and *E*_∞_ = 18.8 meV. The maximum likelihood (*γ*, *E*_1*s*−2*p*_) (Fig. 3b, solid line) matches well with the measurement-based ones (symbols). The calculated temperature-dependent MIR response and the corresponding time shift are based on this phase transition model; its validity is further confirmed by the quantitative agreement with the measured data shown in Fig. 3 and Extended Data Fig. 2.

### Polaritonic effects in bulk CrSBr

The excitonic response of CrSBr is exceptionally strong, inherently generating bulk-exciton-polariton resonances^27^, whose energetics is further controlled by sample thickness through cavity effects. It is important to note that the 2*p*_*a*__/*b*_ states are optically dark and, therefore, do not form polaritons. For our 620-nm-thick sample, multiple polariton modes emerge near the 1*s*-exciton interband resonance. Their photon fraction determines the in-plane dispersion of each polariton (Extended Data Fig. 5a). The lower polaritons (LPs) are located energetically below the 1*s* state and the upper polaritons (UPs) above the 1*s* state. Typically, the energy states of the cavity polaritons become more closely spaced as they approach the energy of the bare 1*s*-exciton resonance. Although polaritonic effects do not qualitatively influence the interpretations discussed in the main text, there are some quantitative corrections. For instance, intraexcitonic transitions exhibit a shift in the *E*_1*s*−2*p*_ resonance energy, which is fully included in the analysis, as discussed above.

### Complementary experiments quantifying polaritonic effects

Extended Data Fig. 5b shows the pump-induced change in the MIR absorption (Δ*α*) and the real part of the dielectric function (Δ*ε*_1_) for different pump–probe delay times *t*_pp_ up to 4 ps and for a pump fluence of *Φ*_NIR_ = 75 μJ cm^–2^. At *t*_pp_ = 0.5 ps, the response is dominated by a maximum in Δ*α* at an energy of 50 meV and a corresponding dispersive feature in Δ*ε*_1_. At later *t*_pp_, the resonance peak in Δ*α* experiences a blueshift *Δ* that saturates at 4 ps and reaches a maximum value of *Δ* = 10 meV (Extended Data Fig. 5a, yellow arrow). For our pump tuned 215 meV above the 1*s*-exciton energy, photoexcitation initially creates unbound electron–hole states, which rapidly form higher-lying lower polaritons close to the bare 1*s*-exciton energy. At early delays, the MIR pulses probe the transition from these states into the 2*p*_*b*_ orbital located 50 meV higher in energy (Extended Data Fig. 5a, light-blue arrow). Within 4 ps, the cavity polaritons will gradually relax towards energetically lower-lying LP states and establish a quasi-equilibrium of the momentum and energy distributions of excitons and polaritons (curved arrow), which increases the energy separation from the 2*p*_*b*_ state. For 1*s*–2*p*_*b*_ transitions driven by the MIR probe transients, only polariton branches with a sufficiently large excitonic character play a role, corroborating the polaritonic energy shift of *Δ* = 10 meV, identified above and in the main text.

To further decouple the polaritonic and excitonic effects in CrSBr, we compare the internal structure of the excitons in a bulk and a thin flake of CrSBr. We prepared a flake with a thickness of 20 nm, in which polaritonic hybridization effects are irrelevant, because the optical path length is substantially smaller than the wavelength of NIR light. Extended Data Fig. 5c shows the pump-induced change in the dielectric response of the bulk (dark blue) and the thin film (red and orange) for the same photoexcitation density. For the relatively high pump fluence (*Φ*_NIR_ = 640 μJ cm^–2^), chosen for improved signal-to-noise ratio, the renormalization of the resonance energy in the bulk sample towards 62 meV already occurs within a delay time of *t*_pp_ = 1 ps. By contrast, the 1*s*–2*p* transition energy in the thin sample is located at 50 meV for the same pump fluence and does not show any shift with delay time. Since the energy difference (10 meV) between the 1*s*–2*p* resonances in the thin and bulk samples exactly matches the ultrafast blueshift *Δ* in the bulk sample due to polaritonic corrections (compare Extended Data Fig. 5a,b), we can conclude that the thin sample remains unaffected by strong light–matter coupling^27^. Consequently, the resonance in the thin sample represents the bare 1*s*–2*p*_*b*_ transition of the exciton in contrast to thicker samples requiring polaritonic corrections.

Furthermore, the temperature-dependent 1*s*–2*p* transition in the 20-nm-thin sample of CrSBr exhibits similar features as observed in the 620-nm-thick bulk crystal (Extended Data Fig. 5d). In particular, the resonance considerably broadens across the magnetic phase transition like that in the 620-nm-thick sample. The main difference lies in the lower signal-to-noise ratio obtained from the thinner sample and the redshift of the 1*s*–2*p* resonance due to the absence of polaritonic effects. For these reasons, the measurements discussed in the main text were recorded on the 620-nm-thick sample.

These findings unequivocally confirm that the bulk crystals of CrSBr induce exciton-polaritons, resulting in a renormalization of the 1*s*–2*p* resonance with an energy correction (*Δ*) of 10 meV at low temperatures. Conversely, at high temperatures, the 1*s*–2*p* resonance is primarily influenced by broadening (*γ*), rendering polaritonic corrections negligible (as discussed above). The measured value of *Δ* has been validated by calculations based on the transfer matrix formalism.

### Fitting of temperature-dependent recombination dynamics of excitons

The decay of the exciton density (Fig. 4a) can be fitted using a biexponential decay function:

$$\Delta E={{A}_{1}{\rm{e}}}^{-t/{\tau }_{1}}+{{A}_{2}{\rm{e}}}^{-t/{\tau }_{2}}.$$

Whereas at higher temperatures, we find finite amplitudes for both *A*_1_ and *A*_2_, *A*_2_ vanishes in the low-temperature AFM phase. The corresponding decay constants are shown in Fig. 4b. The fitted amplitudes *A*_1_ and *A*_2_ are converted into density fractions (Supplementary Section 4 and Fig. 4d). Figure 4 illustrates the ultrafast exciton decay in the absence of a magnetic bias, whereas the corresponding dynamics with a magnetic field of 200 mT along the *b* axis are depicted in Extended Data Fig. 6. The maximum of the pump-induced change in the MIR field transient, $\Delta {E}_{{\rm{MIR}}}^{\max }$, as a function of *t*_pp_, exhibits a fast rise across all temperatures, followed by a temperature-dependent decay on the picosecond timescale. At low temperatures, this decay is best fit by a single exponential (*A*_2_ = 0) with a time constant of *τ*_1_ ≈ 11 ps. As the magnetic order changes (Fig. 4c), a slower-decay component appears. The corresponding intra- and interlayer exciton density fractions are shown in Fig. 4e. Compared with the measurements without an external magnetic field, the slower decay and the corresponding density fraction become apparent at a temperature of *T*′ = 80 K.

## Online content

Any methods, additional references, Nature Portfolio reporting summaries, source data, extended data, supplementary information, acknowledgements, peer review information; details of author contributions and competing interests; and statements of data and code availability are available at 10.1038/s41563-025-02120-1.

## Supplementary information

Supplementary Information Supplementary Figs. 1–5 and Sections 1–4.

## Data Availability

The data that support the plots within this paper are available from the publication server of the University of Regensburg at https://epub.uni-regensburg.de/59755/.
