# Supplementary material for: Controlling Coulomb correlations and fine structure of quasi-one-dimensional excitons by magnetic order
Source: Nat Mater. 2025 Feb 19;24(3):384–90. doi: 10.1038/s41563-025-02120-1 (PMC11879853; doi:10.1038/s41563-025-02120-1)
Supplement: Supplementary file 1 — Supplementary Figs. 1–5 and Sections 1–4. [file 41563_2025_2120_MOESM1_ESM.pdf]

nature materials

Supplementary information https://doi.org/10.1038/s41563-025-02120-1

Controlling Coulomb correlations and fine structure of quasi-one-dimensional excitons by magnetic order

In the format provided by the authors and unedited

Table of contents

1. Sample preparation..2
2. Temperature-dependent exciton dynamics..3
3. Excitonic transitions in the presence of a magnetic field..4
4. Estimating intra- versus interlayer exciton fractions. 6

1. Sample preparation

Thin flakes of a CrSBr crystal were prepared by mechanical exfoliation onto a viscoelastic polydimethyl-siloxane (PDMS) film. Afterwards, the exfoliated bulk sample was transferred onto a diamond substrate. Supplementary Fig. 1a depicts an optical micrograph of the sample used in the main text. The elongated shape of the exfoliated CrSBr flake indicates its crystallographic orientation[1,2]. We determine the thickness of the CrSBr flake to be 620 nm using an AFM line scan (see Supplementary Fig. 1b).

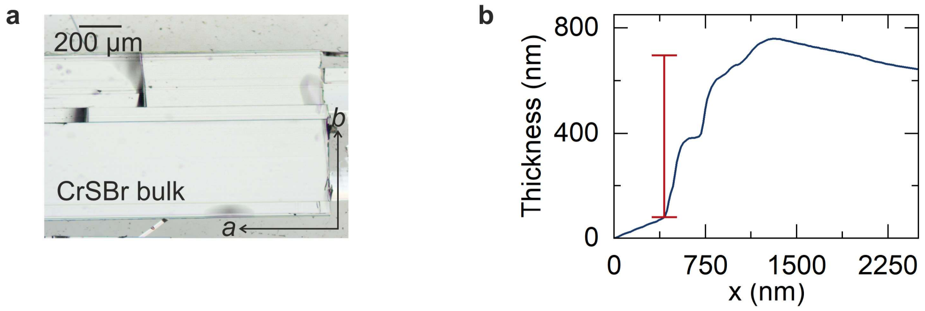

Supplementary Figure 1 | CrSBr sample. a, Optical microscopy image of an exfoliated CrSBr flake on a diamond substrate. The arrows indicate the crystallographic *a*- and *b*-axes. **b,** AFM measurement of the CrSBr sample shown in **a**. The red line indicates a sample thickness of 620 nm.

2. Temperature-dependent exciton dynamics

Figures 3a and c of the main text show the pump-induced changes of the dielectric response and the corresponding MIR waveforms (E_{MIR}), including the simultaneously acquired changes in the electric field (ΔE_{MIR}) at selected temperatures. Supplementary Fig. 2a depicts the full data set for a pump fluence of Φ_{NIR} = 300 µJ/cm^2 at a fixed pump-probe delay time t_{pp} = 4 ps. Whereas in the main text, we focus on the half-cycle marked by the triangle, here we additionally analyze the time shifts t_{shift} for other half-cycles (squares and circles) of ΔE_{MIR} with respect to the reference electric field at a temperature of T = 40 K. The extracted time shifts t_{shift} in Supplementary Fig. 2b exhibit an abrupt jump by several femtoseconds at the Néel temperature[3] T_{N} (vertical dashed line). The inset illustrates how the time shift becomes temperature dependent through the magnetic-order-induced change of effective dimensionality as discussed in the main text.

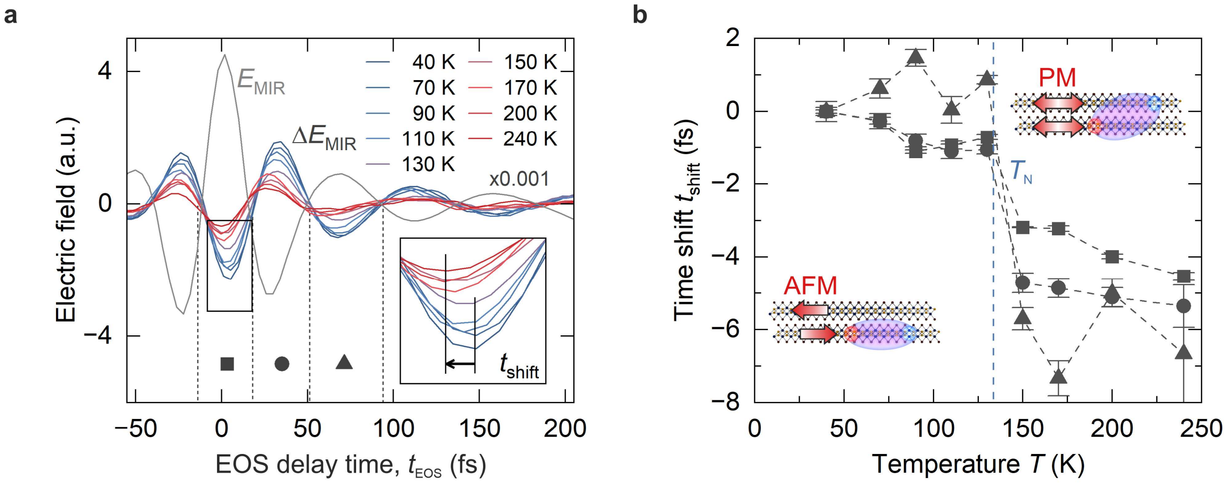

Supplementary Figure 2 | Temperature-dependent dimensionality of excitons. a, Electric field of the MIR probe transient E_{MIR} (scaled by a factor of 10^{-3}) as a function of the electro-optic sampling time t_{EOS}. The simultaneously acquired pump-induced changes to the electric field ΔE_{MIR} are depicted for different temperatures. Inset: Close-up view of the data marked by the rectangle. For decreasing temperature, the minimum of ΔE_{MIR} shifts gradually by a time t_{shift} (arrow). The pump-induced change ΔE_{MIR} at T = 40 K is taken as reference. **b,** Temperature dependence of t_{shift} of the maximum of each oscillation half-cycle (dark grey symbols) of ΔE_{MIR}. The symbols correspond to the oscillation half-cycles marked in **a.** The position of the literature value of the Néel temperature T_{N} is indicated by the blue dashed line. The error bars represent the 95% confidence interval of the fitting procedure used to determine the extrema of the waveforms. Inset: Schematic of the confinement of excitons. Above T_{N}, paramagnetic spin alignment (red double arrows) allows for 3D-like excitons (red and blue spheres connected by the purple ellipsoid). For temperatures below T_{N}, an antiferromagnetic spin order (red arrows) results in 1D-like excitons (purple shading confined to one layer).

3. Excitonic transitions in the presence of a magnetic field

As reported in the main text, the energy of the internal 1*s*–2*p* transition of excitons in CrSBr depends on the magnetic order and the lattice temperature. We repeated the same experiments as in Fig. 3 of the main text with an additional magnetic bias field of $B = 200$ mT applied along the crystallographic *b*-axis. These supporting results are shown in Supplementary Fig. 3, while the key findings are summarized in Fig. 3b,d of the main text. Here, we show the underlying data in the frequency- and time-domain. We recorded the pump-induced changes of the MIR absorption ($\Delta\alpha$, which is directly proportional to $\mathrm{Im}[\xi]$) and the real part of the dielectric function ($\Delta\varepsilon_1$) for temperatures above and below T_N. At low temperatures, the pump-induced response is dominated by a resonance at an energy of 62 meV in accordance to Fig. 3a. However, in contrast to the measurements without an external magnetic field, the maximum in $\Delta\alpha$ disappears already at a temperature of $T' = 80$ K, that is ~50 K below T_N. The abrupt change in time shift t_{shift} (see Supplementary Fig. 3b,c) is similarly shifted to T_N'. As discussed in the main text, an external magnetic field of 200 mT induces an intermediate ferromagnetic phase, whose transition temperature is 50 K (ref. [4,5]) smaller than T_N, affecting the excitonic resonance energy.

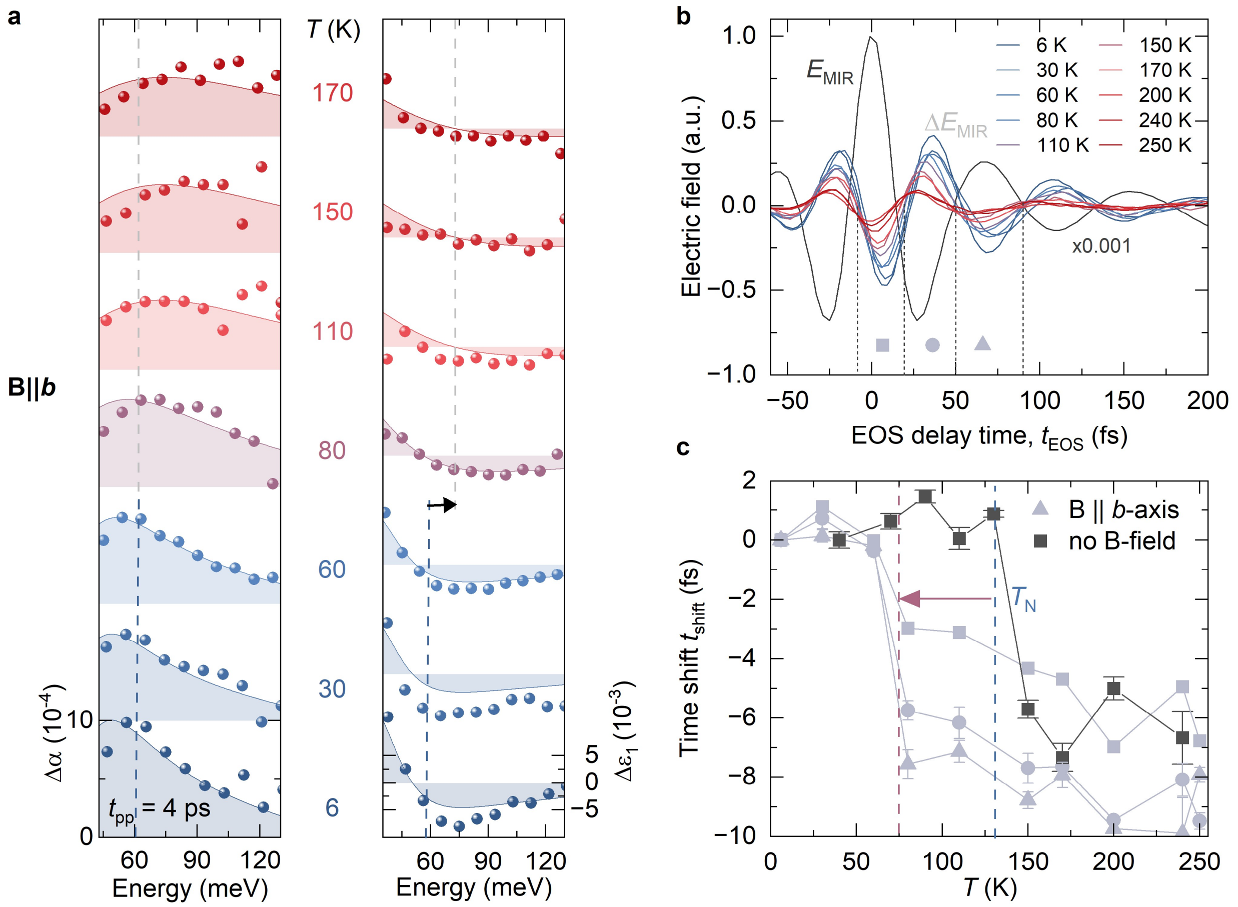

Supplementary Figure 3 | Magnetic field as additional tuning knob. a, Pump-induced change of the MIR absorption ($\Delta\alpha$) and the real part of the dielectric function ($\Delta\varepsilon_1$) as a function of the photon energy at a pump–probe delay time $t_{pp} = 4$ ps for different temperatures with an applied magnetic field along the *b*-axis ($\Phi_{NIR} = 300$ µJ/cm^2). The colour-coded measured (spheres) and computed (shaded areas) data for different temperatures has been vertically offset. The vertical line in the right panel tracks the zero crossing of $\Delta\varepsilon_1$, revealing an abrupt jump by 7 meV. **b,** Electric field of the MIR probe transient E_{MIR} as a function of the electro-optic sampling time t_{EOS}, which has been scaled by a factor of 10^{-3}. The simultaneously acquired pump-induced changes to the electric field ΔE_{MIR} are depicted for different temperatures. For decreasing temperatures, the extrema of ΔE_{MIR} shift gradually by a time t_{shift}. **c,** Time shift t_{shift} of the maximum of each oscillation half-cycle (light grey symbols, compare **b**) of ΔE_{MIR} with respect to $T = 6$ K as a function of the temperature. The symbols correspond to the oscillation half-cycles marked in **b.** For comparison, the time shifts t_{shift} for one half-cycle of ΔE_{MIR} (dark grey rectangles) as a function of the temperature without an applied magnetic field are shown. The position of the Néel temperature T_N is indicated by the blue dashed line. The red dashed line highlights the reduced transition temperature *T*' in the presence of an external magnetic field. The lines connecting the symbols serve as a guide to the eye. The error bars represent the 95% confidence interval of the fitting procedure used to determine the extrema of the waveforms.

4. Estimating intra- versus interlayer exciton fractions.

As demonstrated in Fig. 4, the measured transient pump-induced change of the MIR electric field, $\Delta E(t_{\mathrm{pp}}, t_{\mathrm{EOS}})$, determined at a fixed electro-optic sampling time t_{EOS}, decays with the pump–probe delay time t_{pp}. A straightforward biexponential fit yields two exponentially decaying components $\Delta E_{\mathrm{1L,fit}}(t_{\mathrm{pp}})$ and $\Delta E_{\mathrm{8L,fit}}(t_{\mathrm{pp}})$ as a function of t_{pp}. We have assigned these components to intralayer (1L, single layer) and interlayer (8L, eight layers as assigned in Methods) excitons corresponding to Extended Data Fig. 4. To confirm this assignment independently, we will first estimate the fractions of intra- versus interlayer excitons and then verify whether these fractions agree with complementary measurements presented in Fig. 3 of the main text.

To construct such exciton fractions with minimal assumptions from the measured $\Delta E_{\mathrm{1L,fit}}(t_{\mathrm{pp}})$ and $\Delta E_{\mathrm{8L,fit}}(t_{\mathrm{pp}})$ fits, we start with a simple model based on independent responses of excitons localized to 1L or spread out across 8Ls. We use a broadening parameter $\gamma_{\mathrm{1L}} = 12$ meV to construct the response $\xi_{\mathrm{1L}}(\omega)$ and $\gamma_{\mathrm{8L}} = 110$ meV for $\xi_{\mathrm{8L}}(\omega)$; these choices define the pure 1L and 8L exciton responses, respectively, aligning well with γ extremes in Fig. 3 of the main text. For simplicity, we omit the temperature dependence of both γ_{1L} and γ_{8L} to deduce the computational MIR responses,

$$\xi(\omega) = n_{\mathrm{1L}}\, \bar{\xi}_{\mathrm{1L}}(\omega) + n_{\mathrm{8L}}\, \bar{\xi}_{\mathrm{8L}}(\omega)\,, \tag{2}$$

for any temperature and density combination of intralayer (n_{1L}) and interlayer (n_{8L}) excitons. The normalized quantities $\bar{\xi}_{\mathrm{1L}}(\omega)$ and $\bar{\xi}_{\mathrm{8L}}(\omega)$ are independent of exciton densities, thus, describing intensive excitonic properties.

Following the same procedure as described in Methods, we can convert Eq. (2) into MIR transients,

$$\Delta E(t_{\mathrm{pp}}, t_{\mathrm{EOS}}) = n_{\mathrm{1L}}(t_{\mathrm{pp}})\, \Delta\bar{E}_{\mathrm{1L}}(t_{\mathrm{EOS}}) + n_{\mathrm{8L}}(t_{\mathrm{pp}})\, \Delta\bar{E}_{\mathrm{8L}}(t_{\mathrm{EOS}}), \tag{3}$$

by assuming sufficiently slowly varying exciton densities on the few femtosecond (t_{EOS}) scale compared to the picosecond scale set by the recombination dynamics (t_{pp}). Then the time dependences on t_{pp} and t_{EOS} separate between $n_{\mathrm{1L/8L}}(t_{\mathrm{pp}})$ and $\Delta\bar{E}_{\mathrm{1L/8L}}(t_{\mathrm{EOS}})$. As a result, $n_{\mathrm{1L}}(t_{\mathrm{pp}})\, \Delta\bar{E}_{\mathrm{1L}}(t_{\mathrm{EOS}})$ corresponds to the fitted $\Delta E_{\mathrm{1L,fit}}(t_{\mathrm{pp}}) \equiv A_1 e^{-t_{\mathrm{pp}}/\tau_1}$ component and $n_{\mathrm{8L}}(t_{\mathrm{pp}})\, \Delta\bar{E}_{\mathrm{8L}}(t_{\mathrm{EOS}})$ to the $\Delta E_{\mathrm{8L,fit}}(t_{\mathrm{pp}}) \equiv A_2 e^{-t_{\mathrm{pp}}/\tau_2}$, extracted from measurements (Fig. 4 of the main text) at a fixed delay time t_{EOS}. We simply use Eq. (1) to connect $\Delta E_{\mathrm{1L,fit}}$ and $\Delta E_{\mathrm{8L,fit}}$ with the fit coefficients $A_{1,2}$ and $\tau_{1,2}$. Using Eq. (3) and these identifications, we find a simple conversion formula,

$$\frac{n_{\mathrm{1L}}(t_{\mathrm{pp}})}{n_{\mathrm{8L}}(t_{\mathrm{pp}})} \equiv C(t_{\mathrm{EOS}}) \frac{\Delta E_{\mathrm{1L,fit}}(t_{\mathrm{pp}})}{\Delta E_{\mathrm{8L,fit}}(t_{\mathrm{pp}})}, \text{ with } C(t_{\mathrm{EOS}}) \equiv \frac{\Delta\bar{E}_{\mathrm{8L}}(t_{\mathrm{EOS}})}{\Delta\bar{E}_{\mathrm{1L}}(t_{\mathrm{EOS}})}, \tag{4}$$

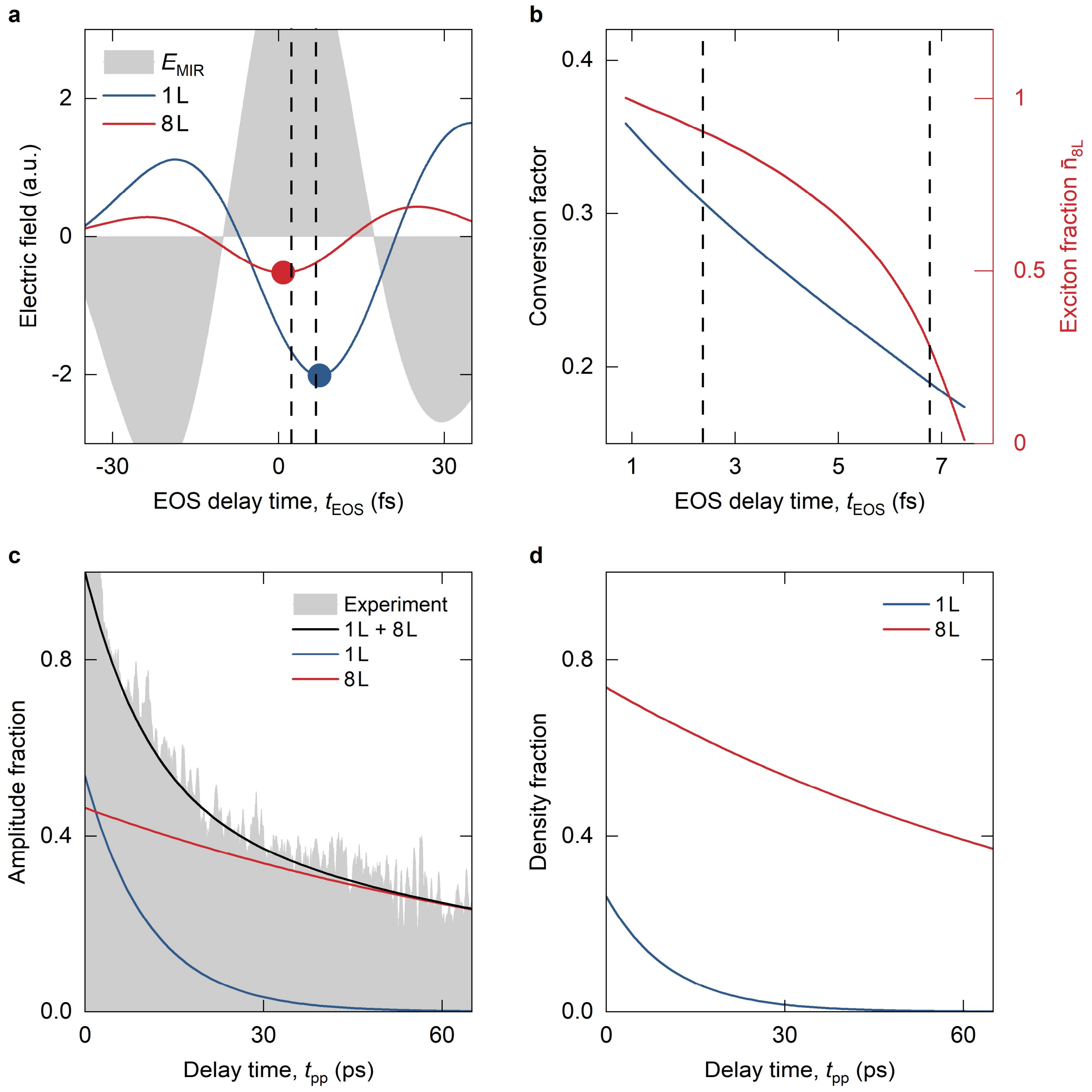

Supplementary Figure 4 | Density conversion. a, Electric field of the MIR reference transient E_{MIR} (grey shaded area) and the pump-induced changes to the field induced by excitons confined in 1L (red line) and spread out across 8L (blue line) as function of t_{EOS}. The exciton dynamics is monitored by measuring the maximum field response at a fixed t_{EOS}. The experimental t_{EOS} range of interest is marked by the vertical dashed lines. **b,** Calculated conversion factor (blue line) between amplitude and density ratios as function of fixed t_{EOS}. Density fractions of 8L excitons (red line) that yield the peak of the $\Delta E(t_{\mathrm{EOS}})$ transient to appear at $t_{\mathrm{peak}} = t_{\mathrm{EOS}}$. **c,** Measured exciton decay dynamics (shaded area) at a temperature of $T = 200$ K is decomposed into contributions from fast decaying excitons, localized to a single layer (1L, blue line) and slow decaying excitons, delocalized over multiple layers (8L, red line). **d**, Estimated density fractions of 1L and 8L excitons based on the conversion formula (5) as a function of the pump-probe delay time t_{pp}.

independent of the excited exciton mixture, and thus t_{pp}.

Supplementary Fig. 4a compares the MIR electric field E_{MIR} (shaded area) with the computed responses $\Delta\bar{E}_{1\mathrm{L}}(t_{\mathrm{EOS}})$ (dark blue line) and $\Delta\bar{E}_{8\mathrm{L}}(t_{\mathrm{EOS}})$ (dark red line) as function of t_{EOS}. We observe that $\Delta\bar{E}_{1\mathrm{L}}(t_{\mathrm{EOS}})$ and $\Delta\bar{E}_{8\mathrm{L}}(t_{\mathrm{EOS}})$ have very different amplitudes and shapes so that the combined time-domain response $\Delta E(t_{\mathrm{pp}}, t_{\mathrm{EOS}})$ must change as a function of exciton fractions, according to Eq. (3). The negative peak of $\Delta\bar{E}_{1\mathrm{L}}(t_{\mathrm{EOS}})$ (dark blue circle) and $\Delta\bar{E}_{8\mathrm{L}}(t_{\mathrm{EOS}})$ (dark red circle) are separated by 6.5 fs, which assigns the maximal t_{peak} shift caused by the exciton species and is the basis of the analysis in Fig. 3 of the main text.

The complementary analysis of Fig. 4 does not follow t_{EOS} but monitors the t_{pp}-dependent decay of $\Delta E_{1\mathrm{L,fit}}(t_{\mathrm{pp}})$ and $\Delta E_{8\mathrm{L,fit}}(t_{\mathrm{pp}})$ parts of the $\Delta E(t_{\mathrm{pp}}, t_{\mathrm{EOS}})$ at a fixed t_{EOS} to deduce the exciton species. With the help of Eq. (3), we obtain a straightforward conversion from the t_{pp}-decay fits to relative and individual exciton fractions,

$$r_{1\mathrm{L},8\mathrm{L}}(t_{\mathrm{pp}}) \equiv \frac{n_{1\mathrm{L}}(t_{\mathrm{pp}})}{n_{8\mathrm{L}}(t_{\mathrm{pp}})} \equiv C(t_{\mathrm{EOS}}) \frac{\Delta E_{1\mathrm{L,fit}}(t_{\mathrm{pp}})}{\Delta E_{8\mathrm{L,fit}}(t_{\mathrm{pp}})}, \qquad \text{for fixed } t_{\mathrm{EOS}},$$

$$\bar{n}_{1\mathrm{L}}(t_{\mathrm{pp}}) \equiv \frac{n_{1\mathrm{L}}(t_{\mathrm{pp}})}{n_{1\mathrm{L}}(t_{\mathrm{pp}})+n_{8\mathrm{L}}(t_{\mathrm{pp}})} = \frac{1}{1+r_{1\mathrm{L},8\mathrm{L}}(t_{\mathrm{pp}})}, \quad \bar{n}_{8\mathrm{L}}(t_{\mathrm{pp}}) \equiv \frac{n_{8\mathrm{L}}(t_{\mathrm{pp}})}{n_{1\mathrm{L}}(t_{\mathrm{pp}})+n_{8\mathrm{L}}(t_{\mathrm{pp}})} = \frac{r_{1\mathrm{L},8\mathrm{L}}(t_{\mathrm{pp}})}{1+r_{1\mathrm{L},8\mathrm{L}}(t_{\mathrm{pp}})}, \tag{5}$$

for 1L and 8L excitons, respectively.

To perform these conversions, we need to know $C(t_{\mathrm{EOS}})$ at each fixed t_{EOS} of the measurement. This computed quantity is shown in Supplementary Fig. 4b as a blue line for t_{EOS} ranging from the peak $\Delta\bar{E}_{8\mathrm{L}}(t_{\mathrm{EOS}})$ at $t_{\mathrm{EOS}} = 2.4$ fs to the peak $\Delta\bar{E}_{1\mathrm{L}}(t_{\mathrm{EOS}})$ at $t_{\mathrm{EOS}} = 6.8$ fs. For this interval, $C(t_{\mathrm{EOS}})$ decays monotonically so that each t_{EOS} assigns a unique $\bar{n}_{8\mathrm{L}}$ fraction that causes the peak of $\Delta E(t_{\mathrm{pp}}, t_{\mathrm{EOS}})$ to occur exactly at the monitored delay time t_{EOS}. We solve Eq. (3) iteratively to find which $\bar{n}_{8\mathrm{L}}$ fraction moves t_{peak} of ΔE to match the fixed t_{EOS}. The resulting $\bar{n}_{8\mathrm{L}}$ curve is shown as a red line (right axis) of Supplementary Fig. 4b; For the studied interval, $\bar{n}_{8\mathrm{L}}$ starts from unity and decays monotonically to zero by the end of the interval.

Although, measuring how the temporal peak t_{peak} of $\Delta E(t_{\mathrm{pp}}, t_{\mathrm{EOS}})$ shifts as function of the pump-probe delay time t_{pp} would allow us to deduce the exciton fractions, it is a prohibitively elaborate approach when we wish to follow exciton dynamics over a large range of pump–probe delays, as is the case in Fig. 4 of the main text. It is experimentally much more feasible to measure the t_{pp} dependence of $\Delta E(t_{\mathrm{pp}}, t_{\mathrm{EOS}})$ at a fixed t_{EOS} to avoid costly, full t_{EOS} scans. As technical steps, we start from the computed $C(t_{\mathrm{EOS}})$ and, at each temperature, from the fitted $\Delta E_{1\mathrm{L,fit}}(t_{\mathrm{pp}})$ and $\Delta E_{8\mathrm{L,fit}}(t_{\mathrm{pp}})$ at a fixed delay time t_{EOS} that corresponds to the measured peak response at $t_{\mathrm{pp}} = 4$ ps, and then apply Eq. (5) to directly convert the fits to density

fractions $\bar{n}_{1L}(t_{pp})$ and $\bar{n}_{8L}(t_{pp})$. Supplementary Fig. 4c illustrates the procedure with the $\Delta E(t_{pp,}t_{EOS})$ data (shaded area) measured at $T = 200$ K, the fit (black line) and its 1L (blue line) and 8L (red line) components. The conversion step uses the computed $C(t_{EOS})$ (from Supplementary Fig. 4b) and Eq. (5). This case yields a sizable interlayer exciton fraction ($\bar{n}_{8L}$, red line in Supplementary Fig. 4d) from the beginning. We also see that the intralayer exciton fraction ($\bar{n}_{1L}$, blue line) decays so that only interlayer excitons remain for times greater than 30 ps. These fractions are shown also in Fig. 4a (top panel) of the main text.

As this assignment is based on measured decay curves, we can validate the density conversion Eq. (5) independently by comparing how well the obtained exciton fractions predict the actually measured t_{peak}, which is the t_{EOS} time when the $\Delta E(t_{pp}, t_{EOS})$ transient peaks. Specifically, the deduced $\bar{n}_{8L}$ also predicts t_{peak} through the red curve in Supplementary Fig. 4b, whereas Fig. 3 of the main text determines t_{peak} directly through t_{EOS} scans at a fixed pump-probe delay time $t_{pp} = 4$ ps. Following the steps in Supplementary Fig. 4 for four representative temperatures, we deduce $\Delta E_{1L,fit}(t_{pp})$ and $\Delta E_{8L,fit}(t_{pp})$ from the decay curves, as shown in Supplementary Fig. 5a. Each case is chosen to have a fixed t_{EOS} which matches the negative peak of the $\Delta E(t_{pp}, t_{EOS})$ transient for $t_{pp} = 4$ ps. The typical range of the delay time t_{peak} is summarized in Supplementary Fig. 4b. These fits predict temperature-dependent exciton fractions $\bar{n}_{1L}$ (blue circles) and $\bar{n}_{8L}$ (red triangles), shown in Supplementary Fig. 5b, based on measured decay curves.

As discussed above, we can predict t_{peak} from $\bar{n}_{8L}$ based on t_{pp} decay curves alone. Complementarily, t_{peak} is directly measured (triangles) and computed (solid lines) in Fig. 3d of the main text via the time shift of the peak $\Delta E(t_{pp}, t_{EOS})$ as t_{EOS} is scanned. Supplementary Fig. 5c compares the directly measured t_{peak} (circles) with the computed values of t_{peak} (solid line), and the corresponding values extracted from the t_{pp} decay curves (filled squares). We confirm an excellent agreement between all three data sets, which independently validates the exciton-fraction assignments from the t_{pp} decay analysis.

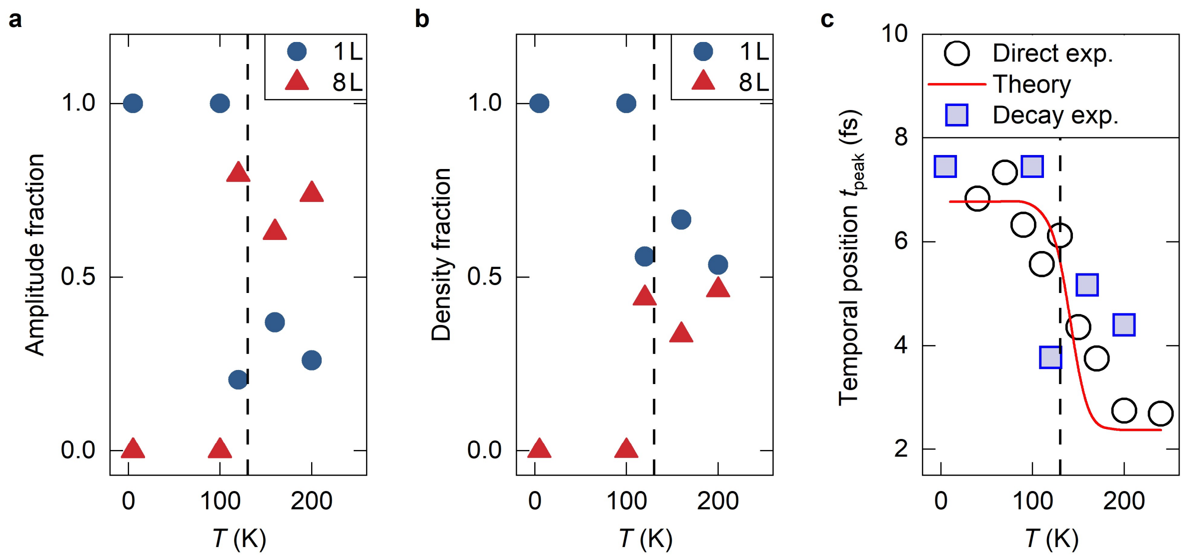

Supplementary Figure 5 | Validating exciton-fraction assignments. a, Fitted probe-response contribution of the 1L (blue circles) and 8L (red triangles) exciton at $t_{pp} = 4$ ps as a function of temperature. The dashed line indicates the critical temperature T_N. **b,** Converted density fraction of the 1L (blue circles) and 8L (red triangles) excitons, following the analysis steps of Supplementary Fig. 4. **c,** Temperature dependence of the time t_{peak}, the point where $\Delta E(t_{pp}, t_{EOS})$ peaks as a function of t_{EOS}. Circles and solid line correspond to the direct t_{peak} measurements and computations of the first oscillation half-cycle, respectively, in Fig. 3 of the main text. The squares follow from converting the t_{pp}-decay-based $\bar{n}_{8L}$ into t_{peak}, using the conversion curve of Supplementary Fig. 4b.

References

1. Telford, E. J. *et al.* Layered Antiferromagnetism Induces Large Negative Magnetoresistance in the van der Waals Semiconductor CrSBr. *Adv. Mater.* **32**, 2003240 (2020).

2. Lee, K. *et al.* Magnetic Order and Symmetry in the 2D Semiconductor CrSBr. *Nano Lett.* **21**, 3511–3517 (2021).

3. Göser, O., Paul, W. & Kahle, H. G. Magnetic properties of CrSBr. *J. Magn. Magn. Mater.* **92**, 129–136 (1990).

4. Long, F. *et al.* Intrinsic magnetic properties of the layered antiferromagnet CrSBr. *Appl. Phys. Lett.* **123**, 222401 (2023).

5. Ye, C. *et al.* Layer-Dependent Interlayer Antiferromagnetic Spin Reorientation in Air-Stable Semiconductor CrSBr. *ACS Nano* **16**, 11876–11883 (2022).
